# A comprehensive comparison of four methods for extracting lipids from Arabidopsis tissues

**DOI:** 10.1186/s13007-020-00697-z

**Published:** 2020-12-03

**Authors:** Cheka Kehelpannala, Thusitha W. T. Rupasinghe, Thomas Hennessy, David Bradley, Berit Ebert, Ute Roessner

**Affiliations:** 1 School of BioSciences, The University of Melbourne, Melbourne, VIC 3010 Australia; 2 Sciex, 2 Gilda Ct, Mulgrave, VIC 3170 Australia; 3 Agilent Technologies Australia Pty Ltd, 679 Springvale Road, Mulgrave, VIC 3170 Australia

**Keywords:** Lipid extraction methods, Arabidopsis, Lipids, Mass spectrometry, QTOF, HPLC, LC–MS, Untargeted lipid analysis

## Abstract

**Background:**

The plant lipidome is highly complex, and the composition of lipids in different tissues as well as their specific functions in plant development, growth and stress responses have yet to be fully elucidated. To do this, efficient lipid extraction protocols which deliver target compounds in solution at concentrations adequate for subsequent detection, quantitation and analysis through spectroscopic methods are required. To date, numerous methods are used to extract lipids from plant tissues. However, a comprehensive analysis of the efficiency and reproducibility of these methods to extract multiple lipid classes from diverse tissues of a plant has not been undertaken.

**Results:**

In this study, we report the comparison of four different lipid extraction procedures in order to determine the most effective lipid extraction protocol to extract lipids from different tissues of the model plant *Arabidopsis thaliana*.

**Conclusion:**

While particular methods were best suited to extract different lipid classes from diverse Arabidopsis tissues, overall a single-step extraction method with a 24 h extraction period, which uses a mixture of chloroform, isopropanol, methanol and water, was the most efficient, reproducible and the least labor-intensive to extract a broad range of lipids for untargeted lipidomic analysis of Arabidopsis tissues. This method extracted a broad range of lipids from leaves, stems, siliques, roots, seeds, seedlings and flowers of Arabidopsis. In addition, appropriate methods for targeted lipid analysis of specific lipids from particular Arabidopsis tissues were also identified.

## Background

Lipids are a large group of highly diverse compounds present in all living organisms and cell types [1]. They play a myriad of crucial roles in biological systems as structural components of membranes [2], for energy storage [3], as signalling molecules in various biological pathways and modulators of cellular functions and diseases [4]. Plant lipids are highly complex [5] and essential for plant growth and development [6]. However, little is known about their composition in different tissue types and related functions. To date, mass spectrometry is the most prevalent technique applied to detect and analyze lipids in biological samples due to its high sensitivity, mass accuracy and scan speed [7].

As a result of recent advancements in mass spectrometry and the development of accompanying lipid identification software, the detection and subsequent identification of many lipid species is now possible. However, attempts to comprehensively characterize the lipidome of a biological system, even while making use of recent advances in mass spectrometry, is futile without the prior application of an efficient lipid extraction method. Thus, the first step for a comprehensive lipid analysis is the efficient extraction of all lipids from a tissue. A weak extraction protocol causes loss of sensitivity, reproducibility, accuracy and precision of the detection, analysis and quantification of any lipid in a sample [8]. The extraction solvent of choice needs to be able to effectively solvate both relatively polar lipids, neutral and non-polar lipids [8, 9]. The extraction procedure must include steps that allow the elimination of particulate matter, reduce chemical and matrix effects and deliver the target compounds in solution at concentrations that are adequate for subsequent detection, quantitation and analysis [8]. Numerous extraction protocols are currently being used to extract lipids from plant tissues. Most of these extraction protocols are adaptations of protocols developed for the extraction of lipids from animal tissues, [10–12] such as the Folch [12] and Bligh and Dyer method [11]. However, these may not be as effective in extracting plant lipids since the lipid composition of plant tissues is unique and different from that of animal tissues [13, 14].

The “gold standard” in lipid biochemistry for the extraction of lipids from animal tissues is the method developed by Folch et al. [12], which uses chloroform (CHCl_3_) and methanol (MeOH) mixed with water [10]. In this method, a biphasic system is generated with an upper phase containing non-lipidic material and a lower phase containing lipidic compounds. Modifications to the protocol published by Folch et al. [12] have led to improved lipid extraction protocols such as the Bligh and Dyer protocol [11] and that by Matyash et al. [10].

Bligh and Dyer [11] found that more lipids can be extracted from frozen fish samples by using a monophasic extraction solvent first before converting it to a biphasic solution. The authors used a mixture of chloroform and methanol (1:2), which, when homogenized with the tissue, mixes with the water in it and forms a monophasic solution. After that, it was diluted with water and chloroform to a final ratio of CHCl_3_, MeOH and water (2:2:1.8) thereby producing a biphasic system with the chloroform layer containing non-polar compounds and the methanol–water layer containing polar compounds [11].

The Bligh-Dyer protocol [11] was tested by de la Roche et al. [15] to extract lipids from wheat *Triticum aestivum* L. cv seeds and the authors found that it is effective in extracting phospholipids but not neutral lipids. This observation led to the hypothesis that the solvent ratios used in the Bligh-Dyer protocol [11] are too polar to extract triglycerides [15]. Consequently, de la Roche et al. [15] added a boiling step with 2-propanol to extract neutral lipids before the application of the Bligh-Dyer protocol [11]. This method was more efficient in extracting lipids from wheat seeds in terms of the complete recovery of total lipids, including total fatty acids and total phospholipids than the application of the Bligh-Dyer protocol alone [15]. Most importantly, boiling the plant tissue with 2-propanol also inactivates lipolytic enzymes such as phospholipases thus preventing lipid degradation [15, 16].

Ryu and Wang [17] simplified the procedure developed by de la Roche et al. [15] by adding CHCl_3_ and water directly to the 2-propanol to extract lipids from leaves of castor bean *Ricinus communis* L. The proportions of solvents remained the same as previously reported by Bligh and Dyer; however, 2-propanol was used instead of MeOH. This was followed by two extraction steps of the plant material with CHCl_3_: MeOH (2:1). A further improvement to the protocol was the addition of 0.01% butylated hydroxytoluene to all extraction solvents, which minimized lipid oxidation [17].

In 2002, Welti et al. [18] adapted the protocol from Ryu and Wang [17] to extract lipids from leaves of Arabidopsis. In contrast to the Ryu and Wang [17] protocol, which included two extraction steps with CHCl_3_: MeOH (2:1), Welti et al. [18] extracted the plant material five times with CHCl_3_: MeOH (2:1). The combined extracts were washed with 1 M potassium chloride (KCl), similar to the Ryu and Wang [17] protocol; however, an additional wash step with water was included [18]. Even though this protocol is elaborate and time-consuming, it has been used extensively to extract lipids from a variety of plant tissues including wheat leaves [19, 19], and Arabidopsis seeds [21] and leaves [22].

A recent publication by Shiva et al. [23] details a simplified version of the multi-step protocol published by Ryu and Wang [17] reducing it to a single extraction step with a 24 h incubation time. In this new protocol, multiple extraction steps and washing of the extract with KCl followed by water have been eliminated. The method has been tested on leaf material from Arabidopsis and *Sorghum bicolour* and proven to provide comparable extraction efficiencies as the methods by Ryu and Wang [17] and Welti et al. [18]. However, a direct comparison of the extraction efficiencies of the protocols detailed by Welti et al. [18] and Shiva et al. [23] on other Arabidopsis tissues, such as flowers, stems, siliques, seeds, roots and seedlings, has not been undertaken.

All the methods mentioned above employ CHCl_3_ as an extraction solvent which is undesirable due to several reasons [10]. Beyond the health hazards associated with the use of CHCl_3_ due to its carcinogenicity, another concern associated with the use of CHCl_3_ is that it decomposes to phosgene and hydrochloric acid; whereby it can induce changes to the structure of some lipid species [10]. Thus, a lipid extraction protocol developed by Matyash et al. [10] replaced the CHCl_3_ used by Folch et al. [12] with methyl-tert-butyl ether to extract lipids from bacteria and mouse brain [10]. This protocol has been later adapted by Hummel et al. [24] to extract lipids from Arabidopsis leaves [24]. A significant weakness of the Folch et al. [12] method is the formation of a biphasic system with the lower CHCl_3_ layer containing lipids and the upper MeOH/water layer containing non-lipidic substances. This biphasic system makes the removal of the MeOH/water layer along with the interfacial fluff extremely difficult and can lead to significant technical errors when dealing with large sample sets [10]. The method by Folch et al. [12] further extracts polar and semi-polar metabolites, starch and proteins along with the lipids from Arabidopsis leaf tissue [24], which is unwanted. A comparison of the efficiency of the Hummel et al. [24] method to extract lipids from Arabidopsis tissues other than leaves is interesting since it appears to be suitable for screening large sample numbers due to its simplicity and the substitution of CHCl_3_ with methyl-tert-butyl ether.

In addition to the methods that focus on exclusively extracting lipids, extraction protocols to investigate many types of compounds including sugars, amino acids, organic acids, chlorophyll, waxes, proteins and RNA along with lipids from a single sample have also been developed [25–27]. One of these methods reported by Burgos et al. [27] could extract for the first time 36-C phosphatidylglycerols and eukaryotic phospholipids with 16:3 acyl chains from Arabidopsis leaves. This method uses a CHCl_3_/MeOH/water (1:2.5:1) mixture and an extraction temperature of 4 °C [27].

When considering the diversity of reported lipid extraction protocols, choosing the best method to extract lipids from plant tissues is a major challenge. A comprehensive comparison of the extraction efficiencies of all the available methods is also tricky due to their large number. Thus, most studies are limited to the comparison of four to six extraction methods to discern which is the best to extract lipids from a particular plant tissue while other tissues are disregarded [15, 28, 29]. A comparison of four lipid extraction methods revealed that a CHCl_3_-MeOH extraction based on Bligh and Dyer protocol was the best to extract lipids from tomato fruits, spinach leaves, fresh mature peas and potato tubers [28]. In this study, all the samples were boiled with 2-propanol or water-saturated *n*-butanol before homogenization in a top-drive blender. Plant samples had then been extracted with four different extraction protocols; (A) a single extraction with a mixture of CHCl_3_/MeOH [28], (B) an extraction with CHCl_3_/MeOH/water followed by extraction with CHCl_3_ and concentrated hydrochloric acid (conc. HCl) [28], (C) a modified Bligh and Dyer extraction similar to method B without conc HCL [30] and (D) an extraction with water-saturated butanol [28]. In another study, de la Roche et al. [15] compared six protocols to extract lipids from wheat seeds; (A) extraction with water-saturated butanol [31], (B) hexane extraction [32], (C) extraction with petroleum ether [33], (D) Bligh-Dyer procedure with CHCl_3_-MeOH–water [34], (E) boiling with 2-propanol followed by Bligh-Dyer procedure with CHCl_3_-MeOH–water [34] and (F) extraction with CHCl_3_-MeOH (2:1) [35]. The authors found that boiling the plant material with 2-propanol followed by the Bligh-Dyer protocol was the most efficient method [15]. In a more recent study by Shiva et al. [23], the extraction protocols of (A) Ryu and Wang which employs a multi-step extraction procedure with 2-propanol/CHCl_3_/MeOH/water [17], (B) single-extraction with CHCl_3_/2-propanol/MeOH/water (30:25:41.5:3.5), (C) single-extraction with 300 mM ammonium acetate replacing water, (D) single-extraction with 300 mM acetic acid replacing water were compared [23] where the lipids in leaf tissues of Arabidopsis and *Sorghum bicolor* were analysed. The study showed that the single-extraction with CHCl_3_/2-propanol/MeOH/water (30:25:41.5:3.5) was comparable to the widely used more labour-intensive multi-step extraction method from Ryu and Wang [17, 23]. A single-extraction procedure using 2:1 parts of chloroform: methanol (v/v) developed by Axelsson and Gentili [29] to extract total lipids from green microalgae was found to be more efficient than three previously reported protocols; (A) the Bligh and Dyer protocol, (B) the Selstam and Oquist [36] and (C) the Folch procedure [29].

While it is desirable to select a method, which can efficiently extract total lipids from different plant tissues, it is arduous considering the diversity of solvent systems and conditions being used in different extraction protocols. In the present study, we investigated the efficiency and reproducibility of the four established lipid extraction methods reported by Welti et al. [18] Burgos et al. [27], Hummel et al. [24] and Shiva et al. [23] to generate total lipid profiles from seven Arabidopsis tissues and to select a suitable high-throughput method for the extraction and comparison of total lipids in those tissues.

## Results

### An overview of the extraction methods compared in this study

To determine an optimal extraction method for large-scale untargeted lipidome studies of Arabidopsis, four different protocols (summarised in Table 1) and seven distinct tissues were compared. The method by Burgos et al. [27] was the shortest, simplest and the least time-consuming protocol with 4 h preparation time while the protocol from Welti et al. [18] was relatively long, time-consuming and laborious. The method by Hummel et al. [24] was also challenging as it required the manual separation of two phases. The protocol reported by Shiva et al. [23] was simple and less labour-intensive; however, required a 24 h extraction incubation period (Table 1).

**Table 1:** A detailed overview of the four lipid extraction protocols used in the present study

Time for the extraction of 25 samples	Burgos et al. [27]	Hummel et al. [24]	Shiva et al. [23]	Welti et al. [18]
Total time	4 h	5 h	4 h + 24 h extraction time	12 h
1 h	Homogenize with 1 ml of CHCl_3_/MeOH/water (1:2.5:1)	Homogenize with 1 ml of MeOH:methyl-tert-butyl-ether (1:3)	Homogenize with 400 µl of 2-propanol with 0.01% BHT	Homogenize with 1 ml of 2-propanol with 0.01% BHT
1 h	Shake for 30 min at 4 °C Spin down for 15 min at 4 °C at 13,200 rpm	Incubate for 10 min in a shaker at 4 °C Incubate for 10 min in an ultrasonication bath at room temperature Add 500 µl of water: MeOH (3:1) Vortex and centrifuge at 13,200 rpm for 15 min	Heat the samples at 75 °C while shaking at 1400 rpm for 15 min Cool to room temperature Add 1.2 ml of CHCl_3_/MeOH/water (30/41.5/3.5, v/v/v)	Add 0.5 ml CHCl_3_ and 0.2 ml water Heat at 75 °C while shaking at 1400 rpm for 15 min
1 h		Remove the upper organic phase containing lipids		Shake for 1 h at 1400 rpm at room temperature
4 h				Centrifuge at 1300 rpm for 15 min Re-extract with 0.3 ml of CHCl_3_/MeOH (2:1) with 0.01% BHT, four times
2 h				Wash the combined extracts once with 0.4 ml of 1 M KCl followed with 0.7 ml of water
24 h			Shake for 24 h at 300 rpm and 25 °C	
2 h	Dry down the organic phase in a SpeedVac	Dry down the organic phase in a SpeedVac	Dry down the solvent in a SpeedVac	Evaporate the solvents by SpeedVac

*BHT* butylated-hydroxy-toluene

#### Lipid profiling of different Arabidopsis tissues

**Fig. 1:**
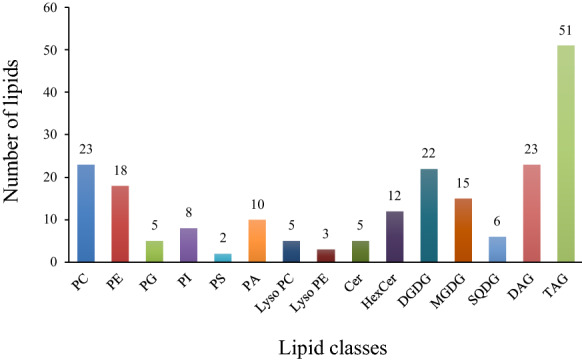
Number of apparent lipids detected and annotated in Arabidopsis leaves. PC: phosphatidylcholine, PE: phosphatidylethanolamine, PG: phosphatidylglycerol, PI: phosphatidylinositol, PS: phosphatidylserine, PA: phosphatidic acid, LPC: lysophosphatidylcholine, LPE: lysophosphatidylethanolamine, Cer-AP: ceramides, HexCer: hexsolyceramides, DGDG: digalactosyldiacylglycerol, MGDG: monogalactosylmonoacylglycerol, SQDG: sulfoquinovosyldiacylglycerol, DAG: diacylglycerol, TAG: triacylglycerol

The untargeted analysis of lipids from Arabidopsis leaf samples yielded 12,274 features, of which 208 lipids were annotated. These lipids belonged to the main lipid classes; sphingolipids, phospholipids, galactolipids and glycerolipids (Fig. 1) and comprised of 23 phosphatidylcholines (PC), 18 phosphatidylethanolamines (PE), 5 phosphatidylglycerols (PG), 8 phosphatidylinositols (PI), 2 phosphatidylserines (PS), 10 phosphatidic acids (PA), 5 lysophosphatidylcholines (LPC), 3 lysophosphatidylethanolamines (LPE), 5 ceramides (Cer),12 hexsolyceramides (HexCer), 22 digalactosyldiacylglycerols (DGDG), 15 monogalactosylmonoacylglycerols (MGDG), 6 sulfoquinovosyldiacylglycerols (SQDG), 23 diacylglycerols (DAG) and 51 Triacylglycerols (TAG) (Fig. 1). Cer, HexCer, PC, PE, PS, LPC and LPE were detected in positive ion mode as [M + H]^+^ adducts. PG, PI, DAG, TAG, DGDG, MGDG and SQDG were detected in positive ion mode as [M + NH_4_]^+^ adducts and PA in negative ion mode as [M-H]^−^ adducts.

Out of the individual lipid species annotated belonging to each of the different classes (Fig. 1), 115 lipid annotations were confirmed by tandem mass spectrometry. The remaining features were annotated by matching experimental *m/z* values with accurate masses of a compiled list of lipids and by aligning their retention times to the identified lipids.

The number of lipids annotated in other Arabidopsis tissues varied from 214 in flowers, 261 in roots, 249 in seedlings, 198 in seeds, 231 in siliques and 257 in stems. A full list of lipids identified from each of the Arabidopsis tissues analyzed in this study is provided in Additional file 1.

#### The four methods showed significant differences in extracting individual lipid classes from different tissues of Arabidopsis

**Fig. 2:**
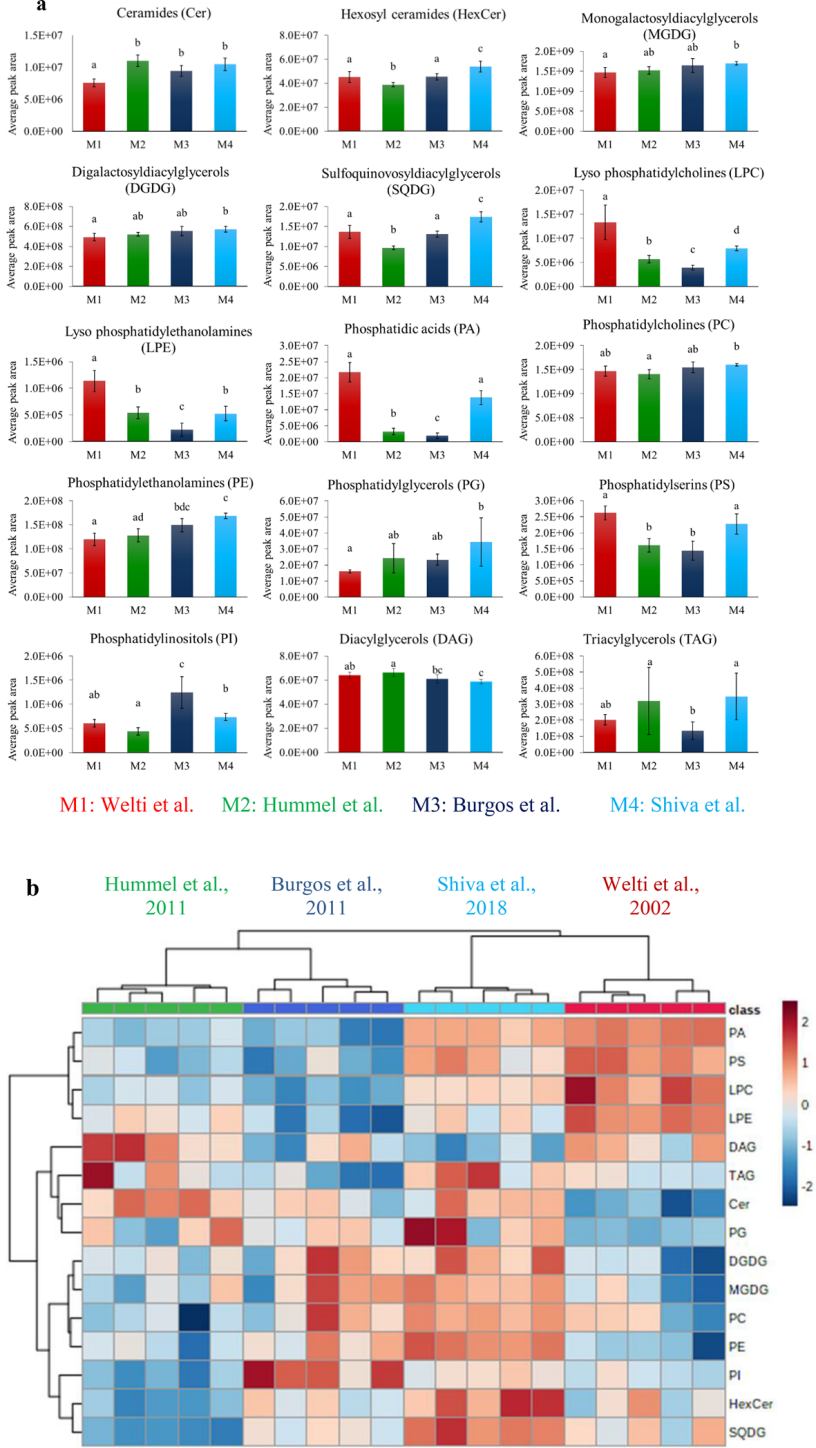
**a** A comparison of the extractability of individual lipid classes from Arabidopsis leaves using the four different lipid extraction methods. Data consist of 208 annotated lipids following LC–MS data processing through MS-DIAL. Bars show the average peak area of the lipids belonging to a class normalized to the fresh weight of the leaf sample (mean ± SD, n = 5). Different letters above bars of the same tissue indicate significant differences (p < 0.05) as determined by ANOVA and Tukey’s test. The bars in red represent the results obtained from the method of Welti et al. [18], green bars represent results from the method of Hummel et al. [24], dark blue bars represent results from the method of Burgos et al. [27] and light blue colour bars represent the results from the method of Shiva et al. [23]. **b.** A heat map of the lipid classes identified in leaf extracts (n = 5) when the four protocols were applied M1: Welti et al. [18], M2: Hummel et al. [24], M3: Burgos et al. [27], M4: Shiva et al. [23]

To determine the effect of the four protocols in extracting individual lipid classes from different Arabidopsis tissues, each lipid class from each Arabidopsis tissue was analysed separately. An ANOVA test followed by Tukey’s test (p < 0.05) confirmed that the four methods showed statistically significant differences between all the analysed lipid classes from leaves of Arabidopsis (Fig. 2a) and several lipid classes from other Arabidopsis tissues (Additional file 2).

#### *The method outlined by Shiva *et al*. showed a high efficiency in extracting lipids from leaves*

In this study and based on the average peak area, the method of Shiva et al. [23] extracted the highest amounts of hexosyl ceramides, MGDGs, DGDGs, SQDGs, PCs, PEs, PGs and TAGs from Arabidopsis leaf tissue (Fig. 2a, b). The Hummel et al. [24] method was the most efficient to extract ceramides; however, this effect was not significantly different (ANOVA followed by Tukey’s test (p < 0.05) to the efficiency obtained using the Shiva et al. [23] protocol. LPEs, LPCs, PSs and PAs were most effectively extracted applying the method by Welti et al. [18] while the protocol from Hummel et al. [24] was the most effective in extracting DAGs (Fig. 2a, b). However, no significant difference in extracting PAs and PSs was observed when the methods of Welti et al. [18] and Shiva et al. [23] were compared. The method by Burgos et al. [27] showed significantly higher efficiency in extracting PIs from Arabidopsis leaves over the other three methods. All four methods showed high repeatability in extracting different lipid classes from leaves, as shown by the hierarchical clustering of the replicates in the heat map (Fig. 2b). A comparison of the total peak areas of all lipids belonging to a specific class showed that except LPEs, PGs and TAGs all the lipid classes were extracted with a coefficient of variation (CV) below 30% by all four methods (Additional file 1). Only the method published by Welti et al. [18] extracted TAGs with a CV below 30%. The repeatability of the four methods was further assessed by comparing the percentage of identified lipids extracted from leaves by each method to the CV below 20% (Table 2). This showed that all four methods extracted ~ 44% of the identified lipids from leaves with a CV lower than 20% (Table 2).

**Table 2:** Determination of the repeatability of the four extraction protocols in extracting lipids from Arabidopsis tissues by comparing the percentage number of identified lipids with the CV value < 20%

	M1 (%)	M2 (%)	M3 (%)	M4 (%)	Highest repeatability
Leaves	44	44	45	44	All
Flowers	45	51	51	60	M4
Siliques	52	29	29	52	M1, M4
Seeds	72	46	36	78	M4
Seedlings	33	35	32	31	M2
Stems	36	16	35	35	M1, M3, M4
Roots	29	29	27	43	M4

M1: Welti et al. [18], M2: Hummel et al. [24], M3: Burgos et al. [27], M4: Shiva et al. [23]

#### *The methods from Burgos *et al*. and Shiva *et al*. were highly efficient in extracting lipids from flowers*

The application of the method reported by Shiva et al. [23] extracted the highest levels of Cer, HexCer, DGDGs, LPEs, PAs, PEs and PSs from Arabidopsis flowers based on the average peak area of lipid classes (Additional file 2: Fig. S1a and b). MGDGs and PCs were best extracted using the protocol of Hummel et al. [24], LPCs using the protocol of Burgos et al. [27] and DAGs using the protocol of Welti et al. [18]. No statistically significant differences in extracting Cer, HexCer, MGDGs, DGDGs, LPCs, LPEs, PCs, PEs and PSs from flowers could be observed between the methods by Burgos et al. [27] and Shiva et al. [23]. However, the Burgos et al. [27] protocol yielded significantly lower amounts of TAGs compared to the other three methods while the protocol of Shiva et al. [23] was least efficient in extracting DAGs. All four methods provided repeatable results among the five independent replicates tested as shown in the heat map (Additional file 2: Fig. S1b). The method from Shiva et al. [23] extracted 60% of the identified lipids from flowers with a CV below 20% while with the method from Welti et al. [18] 45% could be extracted with a CV below 20%. The other two methods both extracted 51% of the identified lipids from flowers with a CV lower than 20% (Table 2).

#### *Lipids from siliques were most efficiently extracted by the Shiva *et al*. method*

In our study, no significant differences were observed between the four methods in extracting DGDGs, PEs, PGs and PCs from siliques (Additional file 2: Fig. S2a). Based on the average peak area, the method of Shiva et al. [23] captured the highest levels of Cer, HexCer, SQDGs, LPCs, PAs and TAGs. The protocol by Hummel et al. [24] extracted the most DAGs; however, its efficiency was not significantly different from the Shiva et al. [23] protocol. The repeatability of the Hummel et al. [24] and Burgos et al. [27] protocols was relatively low across the five replicates analyzed in this study, which is apparent when visualising the data in a heat map (Additional file 2: Fig. S2b). Both methods extracted only 29% of the identified lipids from siliques with a CV below 20% (Table 2). By contrast, the procedures described by Shiva et al. [23] and Welti et al. [18] resulted in highly repeatable data of all lipid classes (Additional file 2: Fig. S2b) with both methods extracting 52% of the identified lipids from siliques with a CV below 20% (Table 2).

#### *The methods by Burgos *et al*. and Shiva *et al*. efficiently extracted lipids from seeds*

The application of the four different methods to dry seeds did not reveal statistically significant differences in extracting DGDGs and PAs (Additional file 2: Fig. S3a). Based on the average peak area, MGDGs, DGDGs and PCs and LPCs could be best extracted with the protocol from Burgos et al. [27]. However, its efficiency in extracting these four lipid classes was not significantly different from that of Shiva et al. [23]. The Burgos et al. [27] protocol was, however, significantly less efficient in extracting DAGs and TAGs from seeds when compared to the other methods. For the extraction of lipids from dry seeds, all methods showed repeatable results except for the method by Welti et al. [18], which yielded an outlier, as shown in the heat map analysis (Additional file 2: Fig. S3b). However, the method of Shiva et al. [23] provided the most reproducible results, extracting 78% of the identified lipids in seeds with a CV below 20% (Table 2).

#### *Lipids from seedlings were best extracted using the Shiva *et al*. and Welti *et al*. protocols*

The four methods did not differ statistically significantly in extracting DGDGs, PCs, PEs and PGs from Arabidopsis seedlings (Additional file 2: Fig. S4a). Based on the average peak area, the protocol reported by Welti et al. [18] best extracted LPCs, HexCer, MGDGs and PAs, while the protocol by Hummel et al. [24] yielded the most DAGs. However, the extraction efficiencies did not vary significantly between the Shiva et al. [23] and Welti et al. [18] protocols in extracting LPCs, HexCer and MGDGs. Cer, SQDGs, PSs and TAGs also were best extracted by the protocol of Shiva et al. [23] (Additional file 2: Fig. S4a). For material from seedlings, only the protocols of Shiva et al. [23] and Welti et al. [18] produced repeatable results for the tested five replicates which can be seen in the heat maps (Additional file 2: Fig. S4b). All four methods extracted 31–35% of the identified lipids from seedlings with a CV below 20% (Table 2).

#### All methods were efficient in extracting lipids from stems

Based on the average peak area, DGDGs and PCs were best extracted from stems by the protocol of Burgos et al. [27], SQDGs by the method of Shiva et al. [23], LPCs and PAs by the method of Welti et al. [18] and PSs and DAGs by the method of Hummel et al. [24]. However, the four methods did not show statistically significant differences for the extraction of Cer, HexCer, MGDGs, PEs, PGs, PIs and TAGs from stems (Additional file 2: Fig. S5a). The repeatability was good for three methods; however, the data obtained using the Shiva et al. [23] protocol lacked repeatability as can be seen in the heat map (Additional file 2: Fig. S5b). A comparison of the percentage of identified lipids with a CV value below 20% from stem material shows that the repeatability was similar for the methods of Welti et al. [18], Shiva et al. [23] and Burgos et al. [27] with 35–36% of the identified lipids captured (Table 2). However, only 16% of the identified lipids in stems extracted by the Hummel et al. [24] method had a CV lower than 20% (Table 2).

#### Lipids from roots can be efficiently extracted with all four methods

When we analyzed Cer, HexCer, MGDGs, DGDGs, SQDGS, LPCs, LPEs, PCs, PEs, PGs and TAGs from roots, no statistically significant differences between the four methods could be observed (Additional file 2: Fig. S6a). However, the four methods extracted PIs, PSs and DAGs with different efficiencies. While the method by Hummel et al. [24] was significantly less efficient in extracting PIs and PSs in comparison to the other methods, its extraction efficiency was high in extracting DAGs. The Hummel et al. [24] protocol was also the only one delivering repeatable data for all lipids as shown by hierarchical clustering and heat map (Additional file 2: Fig. S6b). However, the method of Shiva et al. [23] captured 43% of the identified lipids from roots with a CV below 20% while the other three methods extracted only 27–29% (Table 2).

#### The best method to extract different lipid classes from diverse Arabidopsis tissues

The lipid extraction protocols applied in this study varied in terms of which lipids they extracted best from the different Arabidopsis tissues (Table 3).

**Table 3:** Overview of the most efficient protocols to extract specific lipid classes from different tissues of Arabidopsis

Lipid class	Leaves	Flowers	Siliques	Seeds	Seedlings	Stems	Roots
Cer	M2, M3, M4	All	M3, M4	M2, M3, M4	M1, M2, M4	All	All
HexCer	M4	M1, M3, M4	M2, M3, M4	M1, M4	M1, M2, M4	All	All
LPC	M1	M3, M4	M4	M3, M4	M1, M3, M4	M3, M4	All
LPE	M1	M3, M4	ND	M1, M3, M4	ND	ND	All
DGDG	M2, M3, M4	M3, M4	All	All	All	M1, M3, M4	All
MGDG	M2, M3, M4	M2, M3	ND	M2, M3, M4	M1, M3, M4	All	All
SQDG	M4	ND	M4	M4	M4	M1, M4	All
PA	M1, M4	M1, M4	ND	All	M1	M1, M2, M4	ND
PC	M1, M3, M4	M2, M4	All	M3, M4	All	M1, M3	All
PE	M3, M4	M2, M3, M4	All	All	All	All	All
PG	M2, M3, M4	ND	All	ND	All	All	All
PI	M3	ND	M3	ND	ND	All	M1, M3, M4
PS	M1, M4	M3, M4	M1, M3, M4	M1, M3, M4	M3, M4	M2, M4	M1, M3, M4
DAG	M1, M2	M1, M2	M1, M2, M4	M1, M2, M4	M2	M2	M2, M3
TAG	M2, M4	M1, M2, M4	M1, M2, M4	M1, M2, M4	M1, M2, M4	All	All

The methods which did not statistically significantly differ in their extraction efficiencies are provided together. M1: Welti et al. [18], M2: Hummel et al. [24], M3: Burgos et al. [27], M4: Shiva et al. [23]. *PC* Phosphatidylcholine, *PE* phosphatidylethanolamine, *PG* phosphatidylglycerol, *PI* phosphatidylinositol, *PS* phosphatidylserine, *PA* phosphatidic acid, *LPC* lysophosphatidylcholine, *LPE* lysophosphatidylethanolamine, *Cer-AP* ceramides and *HexCer* hexsolyceramides, *DGDG* digalactosyldiacylglycerol, *MGDG* monogalactosylmonoacylglycerol, *SQDG* sulfoquinovosyldiacylglycerol, *DAG* diacylglycerol, *TAG* triacylglycerol, *ND* Not detected or data inconsistent

The application of the method by Shiva et al. [23] successfully extracted Cer, HexCer, SQDGs, PCs, PEs, PGs, PSs and TAGs from all Arabidopsis tissues analyzed in this study. This method was also the most effective in extracting PAs and MGDGs from most tissues except seedlings and flowers, respectively. However, it was much less efficient in extracting DAGs from leaves, flowers, seedlings, stems and roots. This observation contrasts with what was observed for extractions with the protocol from Hummel et al. [24] which was highly efficient in extracting DAGs and TAGs from all tissues. The method of Burgos et al. [27] was ideal for extracting phospholipids from most tissues but significantly less efficient than the other methods in extracting DAGs and TAGs from any tissue except roots and stems.

#### *The method by Shiva *et al*. is the best suited for the comparison of lipid profiles across different Arabidopsis tissues*

**Fig. 3:**
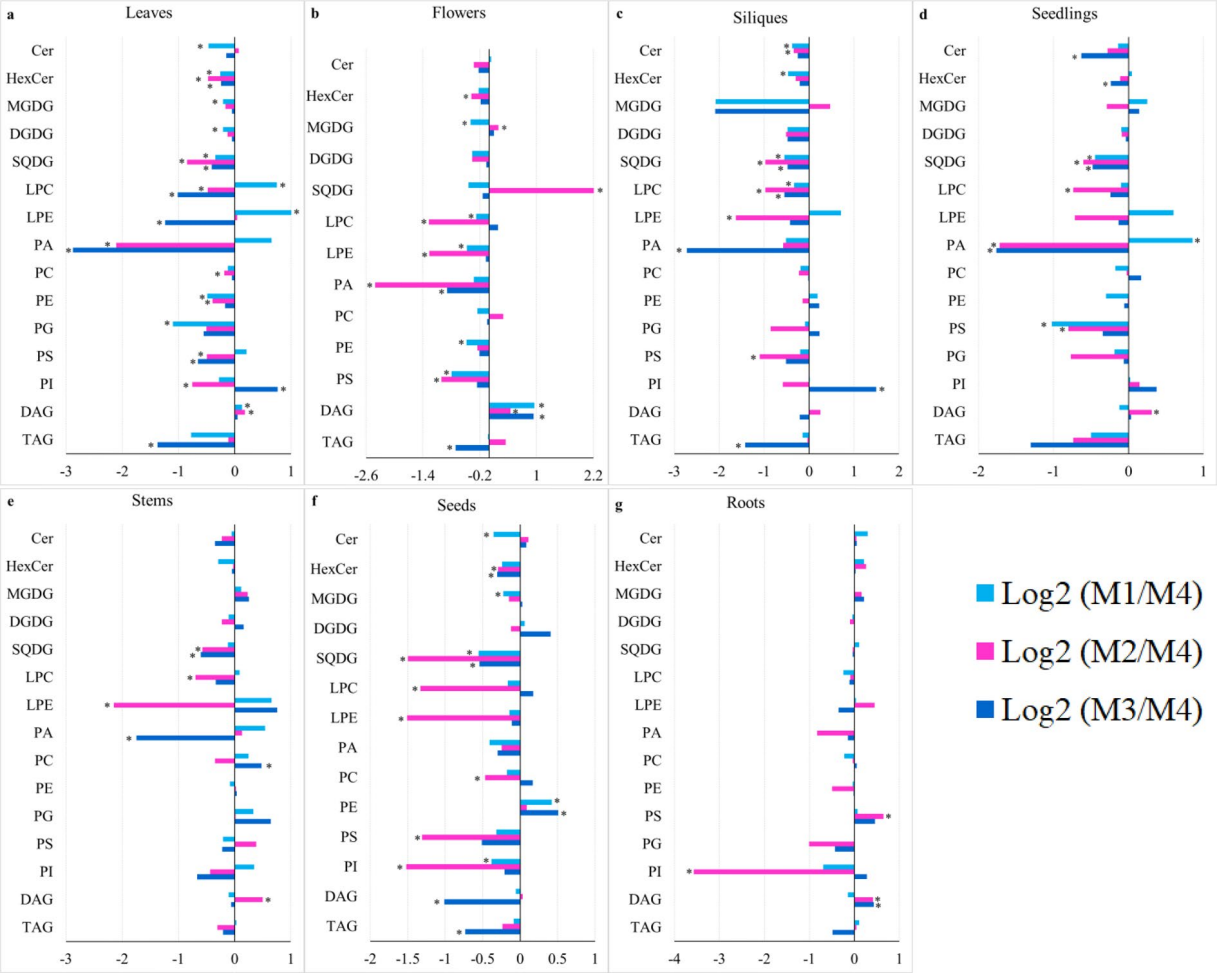
Log2 fold change comparison of lipid classes extracted by the method of Shiva et al. [21] and the methods of Welti et al. [16], Hummel et al. [22] and Burgos et al. [25] (n = 5). Fold changes were calculated by dividing the average peak area of a lipid class extracted by each of the methods Welti et al. (M1), Hummel et al. (M2) and Burgos et al. (M3) by that of Shiva et al. (M4), and then log2 transformed. Significant differences were determined by ANOVA, followed by Tukey’s test (p < 0.05)

The focus of this study was to determine a high-throughput and robust method which can effectively extract total lipids belonging to a wide range of lipid classes and hence allowing the comprehensive profiling of lipids in diverse Arabidopsis tissues. Overall, our study revealed that the application of the method by Shiva et al. [23] successfully extracted all the lipid classes from different tissues of Arabidopsis in a decidedly consistent manner. It is also a simple and straightforward and readily applicable protocol. To further investigate the effectiveness of this method, a fold change analysis comparing the lipid levels extracted by Shiva et al. [23] and the other three methods was carried out (Fig. 3a–g). We found that the method by Welti et al. [18] was significantly more efficient in extracting LPCs from leaves (Fig. 3a), DAGs from leaves and flowers (Figs. 3a, b), PEs from seeds (Fig. 3f) and PAs from seedlings (Fig. 3d) when compared to the method of Shiva et al. [23]. The application of the protocol from Burgos et al. [27] yielded significantly higher amounts of PIs from leaves and siliques (Fig. 3a, c), DAGs from flowers (Fig. 3b), PEs from seeds (Fig. 3f), PCs from stems (Fig. 3e) and DAGs from roots (Fig. 3g) than the Shiva et al. [23] method, while the Hummel et al. [24] method was more efficient in extracting DAGs from leaves (Fig. 3a), flowers (Fig. 3b), seedlings (Fig. 3d), stems (Fig. 3e), roots (Fig. 3g) and SQDGs and MGDGs from flowers (Fig. 3b). In all other cases when we used the method by Shiva et al. [23], we observed either significantly higher extraction efficiencies or no significant difference compared to the three other methods (Fig. 3a–g).

## Discussion

Although numerous analytical methods exist for the extraction of specific lipids, a universal lipid extraction procedure is required to obtain a comprehensive profile that allows screening for a variety of lipids simultaneously. This trait is especially important in an untargeted lipidomic approach to ensure that as many lipids as possible are extracted for subsequent delivery to the solvent system. Untargeted lipid profiling offers a more comprehensive approach when compared to targeted lipid analysis. It can provide intriguing new insights into a sample matrix, for example, through the detection of novel lipids while at the same time allowing to compare many known features [5]. The effective extraction of total lipids from a given tissue sample is the first step that is required to achieve this. Several factors must be considered when selecting an optimal method for the extraction of total plant lipids. The lipid class of interest, reproducibility of the method, ease and rapidity, cost-effectiveness in large scale or routine lipidomic analysis, sample recovery and effective removal of interferents are important factors [7, 37].

This study aimed to determine the most effective method to extract lipids from different tissues of Arabidopsis for an untargeted lipid analysis out of four established lipid extraction methods reported by Welti et al. [18], Burgos et al. [27], Hummel et al. [24] and Shiva et al. [23]. These four extraction protocols have been predominantly used to extract lipids from leaf tissue, and a detailed comparison of how efficient they are in extracting lipids from a variety of Arabidopsis tissues has not been undertaken so far. The method by Welti et al. [18] was used to extract lipids from Arabidopsis leaves to understand changes in membrane lipid profiles in response to cold and freezing stresses [18]. Burgos et al. [27] studied the changes of the glycerolipidome of Arabidopsis leaves in response to temperature and light [27], while Hummel et al. [24] profiled the polar and non-polar lipids from Arabidopsis leaves using ultra-performance liquid chromatography coupled to high-resolution mass spectrometry [24]. Shiva et al. [23] reported a lipid extraction protocol for Arabidopsis and Sorghum leaves which is a modification of the multi-step method used by Ryu and Wang [17] similar to the one used by Welti et al. [18].

Here, these methods were applied as published previously with a minor modification to the protocols from Welti et al. [18] and Shiva et al. [23] where we added a tissue homogenizing step at the start of the extraction. These two protocols did not contain a tissue homogenization step and lipids were directly extracted from intact tissue. Hummel et al. [24] used a Retsch mill (MM301, Retsch, Düsseldorf, Germany) for tissue homogenization, while Burgos et al. [27] used a cryogenic grinding robot. In this study, the plant material was ground to a fine powder in liquid nitrogen using a mortar and pestle followed by cryo-milling using a Precellys tissue homogenizer. This sort of cell disruption allows the extraction solvent to better access and solubilize the lipids [29, 38]. It has been reported that plant material likely needs to be homogenized to a particle size of 300 µm or smaller due to its rigidity to assist in releasing intracellular lipids [29].

The lipid extracts were then analyzed by LC–MS using a high-resolution Agilent QToF 6545 which allowed annotation of lipids based on accurate mass and retention time coupled with data processing through MS-DIAL. Together this approach facilitated the annotation of more than 200 lipids belonging to a broad range of lipid classes from Arabidopsis tissues and allowed the statistical evaluation of the lipid extractability from each tissue by the four extraction protocols.

Based on our observations followed by statistical analysis of the data, the optimal method/s for the extraction of each specific lipid class from different Arabidopsis tissues was determined (summarized in Table 3). We observed that particular methods extract the analyzed lipid classes in different Arabidopsis tissues with varying efficiencies. One reason for the differences in extraction efficiencies could be the variation of individual lipids and their respective amounts in these tissues. The highest amounts of polar lipids normalized to dry organ weight are present in leaves and flowers while roots have the least [39]. This diversity of lipids, their relative amounts in particular tissues and preferences for certain solvents may give rise to substantial differences in their extractability by different methods. Another reason for the observed differences in extraction efficiency could be the mixing of tissue water with the extraction solvents thereby forming a monophasic extraction system [29] which might change the standard solvent ratios given in a protocol. This can lead to inconsistent extraction efficiencies by the same protocol for the same lipid classes from different tissues.

The importance of tissue water content in lipid extraction protocols is well documented, for example, Bligh and Dyer [11] reported that optimum lipid extraction occurs when tissue water is mixed with CHCl_3_-MeOH system to form a monophasic system. The optimum amounts of CHCl_3_ and MeOH should be determined by constructing a phase diagram [11]. Fishwick et al. [28] found that lipids in tissues with high water content are efficiently extracted by CHCl_3_-MeOH systems while being poorly extracted by water-saturated butanol. However, no difference was observed when the extraction efficiency of the two systems was compared using tissues with low water content [28]. Axelson and Gentili [29] reported that increasing the solvent-to-sample ratio makes the extraction system stronger, thereby allowing for more variation in sample content and its size [29]. In this study, the tissue water content has not been accounted for as the focus was to compare the efficiencies of the methods which are already in use to extract lipids from Arabidopsis. As it is apparent that water content is variable among tissues, this may have led to a substantial variation of the extraction efficiencies between the four protocols.

We also observed striking differences in the extractability of certain lipid classes by the four methods. It is reported that TAGs are almost completely soluble in CHCl_3_ with the solubility decreasing when mixed with MeOH. The presence of water in CHCl_3_-MeOH system further decreases the solubility of apolar lipids [40]. We observed that the method by Hummel et al. [24] was the most efficient in extracting DAGs and TAGs from all tissues while the method by Burgos et al. [27] showed poor extractability of these two lipid classes. However, the Burgos et al. [27] method was highly effective in extracting phospholipids from all tissues. The extraction protocol outlined by Burgos et al. [27] uses CHCl_3_, MeOH and water as extraction solvents, and de la Roche et al. [15] has suggested that CHCl_3_, MeOH and water system may be too polar to solubilize TAGs effectively [13]. De la Roche et al. [15] have also observed that the Bligh-Dyer [11] protocol employing a CHCl_3_, MeOH and water (1:2:0.8, v/v/v) system, extracts phospholipids efficiently but not neutral lipids [15].

In addition to DAGs and TAGs, the biphasic extraction method of Hummel et al. [24] also extracted lipids belonging to the major lipid classes such as PC, PE, MGDG and DGDG from several Arabidopsis tissues with good efficacy. Matyash et al. [10] have reported that the biphasic extraction method produces similar or slightly higher recoveries of major lipid classes as the Bligh and Dyer method [10]. However, the Hummel et al. [24] protocol requires the separation of two phases by suction which needs considerable manual input and thereby presents a challenge for large scale lipidomic studies and introduces a significant technical error affecting the reproducibility.

In line with previous reports [23], the methods by Welti et al. [18] and Shiva et al. [23] led to comparable results in our study. However, the multi-step extraction protocol published by Welti et al. [18] was found to be time-consuming and laborious (Table 1). The Welti protocol involved several steps of manual sample manipulation which significantly increased the possibility of human error, and ultimately affected the reproducibility of the results. Due to the lengthy nature of this extraction protocol, it is impractical to use it in routine lipidomic analyses where large sample batches need to be analyzed. In contrast, the protocol by Shiva et al. [23] is shorter and less complex [23]. Although this modified protocol employs an incubation period of 24 h, it is considerably less labour-intensive (Table 1). This allows to obtain extracts from multiple samples and replicates simultaneously.

Because of the lack of commercial standards for many lipid species [41] and practical limitations in generating hundreds of calibration curves for each sample batch [42] untargeted LC–MS based lipidomic studies largely rely on comparative studies, i.e. the comparison of lipid profiles from control and treatment, to arrive at conclusions [5, 43–45]. As a result, it is highly important to test the repeatability of analytical workflows and the applied extraction protocols [43]. We found that ~ 96% of the lipids detected in pooled biological quality control samples had a CV value below 30% (Additional file 1) signifying the consistency of the instrument performance over time. Many studies report CV values lower than 30% to be indicative of the quality of an experiment [5, 46–48].

We analysed the repeatability of the four methods within five technical replicates by comparing the percentage of lipids extracted to the CV below 20%, which is a value widely accepted in the area of biomarker analysis [43, 49]. Overall, the method by Shiva et al. [23] was found to be the most repeatable in extracting lipids from all Arabidopsis tissues as it extracted most of the lipid classes from all Arabidopsis tissues with a CV below 30% (Additional file 1), showed high repeatability as can be seen in hierarchical clustering analysis using heat maps (Fig. 3) and extracted a high percentage of the identified lipids with a CV below 20% (Table 2).

All four methods yielded results comparable with previous studies. For example, we observed that the protocol detailed by Shiva et al. [23] is highly efficient in extracting PAs. This is supported by the Shiva et al. [23] study, where the authors saw an improved recovery of PAs when they compared their method to the widely used extraction protocol from Ryu and Wang [17]. Burgos et al. [27] repeatably extracted most phospholipids present in Arabidopsis leaves. In our study we also noted that the Burgos et al. method [27] is highly efficient in extracting phospholipids (Table 3, Fig. 2 and Additional file 2). Hummel et al. [24] reported that their method is highly efficient in extracting both polar and non-polar lipids from Arabidopsis leaves. This was confirmed in our study where the Hummel et al. [24] protocol efficiently extracted both non-polar lipids such as DAGs and TAGs as well as relatively polar lipids such as phospholipids and galactolipids. Consequently, all methods seem to deliver reproducible results across different laboratories.

It is important to note that previous studies have shown chloroform/methanol mixtures can be inefficient in extracting some plant sphingolipids such as glycosyl inositol phosphoryl ceramides (GIPCs) [23, 50, 51]. Therefore, to investigate sphingolipids, particularly GIPCs, a targeted approach as detailed by Markham and Jaworski [51] is recommended and often used to analyze GIPCs [52, 53]. Markham and Jaworski [51] successfully measured 168 sphingolipids including GIPCs from Arabidopsis leaf samples using a mixture of isopropanol, hexane and water as extraction solvent which they found to be most successful in solubilizing GIPCs [44]. To reduce the interference from other lipids during mass spectrometric detection, Markham and Jaworski treated lipid extracts with monomethylamine, which hydrolyses ester-containing lipids such as phospholipids while sphingolipids remain intact.

Overall, the application of the single-step extraction protocol successfully extracted most of the individual lipid classes from all Arabidopsis tissues with high efficiency and repeatability when compared to the other methods. Thus, its application can be recommended to extract diverse lipid classes from various Arabidopsis tissues for comprehensive lipid profiling.

## Conclusions

Many extraction methods exist to isolate lipids from plant tissue. In this study, a comparison of four popular and widely used protocols was undertaken. The application of these methods to extract total lipids from a variety of Arabidopsis tissues revealed that a single-step protocol with a 24 h extraction period was the most efficient, straightforward and cost-effective. This method was suitable for the extraction of phospholipids, galactolipids, ceramides, diacylglycerols and triacylglycerols from different tissues of Arabidopsis in a highly efficient and reproducible manner. Thus, we recommend this method to extract total lipids from diverse tissues of Arabidopsis for comprehensive and comparative analyses of its lipid content.

## Materials and methods

### Chemicals

All the chemicals used were of the highest purity or analytical grade. All organic solvents were of HPLC grade (Fisher Chemical, USA). Deionized water was produced with a Millipore Milli-Q system (Billerica, MA, USA).

### Plant material and growth conditions

Seeds of wild-type *Arabidopsis thaliana* accession Columbia-0 were obtained from the Arabidopsis Biological Resource Center (ABRC).

#### Flowers, stems, siliques and seeds

To obtain flower, stem, silique and seed material, seeds were placed on peat pellets and vernalized at 4 °C for 3 days. Next, the trays were placed in a growth chamber under a 18/6 h day/night regime at 21 °C [54] and 18 °C (night) temperatures with a daytime light intensity of 100–120 µE and 70% relative humidity.

At two developmental stages described by Boyes et al. [55] plant material was harvested. The first 4–6 leaves were harvested from 28 days old (stage 3.90) pre-flowering plants. Siliques, flowers and stems were obtained from 49 days old (stage 6.90) plants. After harvesting, the plant material was immediately frozen in liquid nitrogen and stored at − 80 °C until further use. Dry seeds were harvested from mature plants 64 days after germination.

#### Seedlings

To obtain seedlings seeds were surface sterilized by washing them with 1 ml of 70% ethanol for 5 min followed by 1 ml of sodium hypochlorite for 10 min under constant shaking at room temperature. The seeds were thoroughly rinsed five times with 1 ml of sterile MilliQ water and plated on Petri dishes (90 × 15 mm) containing sterile solid ½ MS medium. The ½ MS medium contained of 0.5% Murashige and Skoog (1962) mineral salts (PhytoTechnology Laboratories, US), 0.05% of MES hydrate (Sigma), 1% (w/v) sucrose (Sigma) and 0.7% agar (Sigma). The pH was adjusted to 5.6–5.8 with 1 N potassium hydroxide. The seeds were cold stratified for 3 days at 4 °C before they were placed in a growth chamber under 18/6-h day/night cycles at 21 °C (day) and 18 °C (night) temperatures, light intensity of 120 µEm^2^s^−1^ and relative humidity 70%. Seedlings were harvested after 14 days (stage 1.04) and immediately frozen in liquid nitrogen and stored at -80 °C until further use.

#### Roots

To prepare root material, Arabidopsis plants were grown in liquid medium using a system described by Conn et al. [54]. The germination medium and standard growth medium were prepared according to the instructions given by Conn et al. [54]. The method to prepare the germination medium and growth medium is given in Additional file 3. The lids of microcentrifuge tubes were punctured with a needle to form a 1.2–1.8 mm diameter hole in the middle of each lid. The lids were cut off from the tubes and placed on adhesive tape with the tape covering the holes. Each lid was then filled with the germination medium such that a dome is formed while ensuring the medium does not overflow and allowed to solidify. Then, the lids were removed from the tape and placed on the racks of 1 ml micropipette tip boxes filled with the liquid germination medium such that the plug of agar in each lid is in contact with the liquid medium. Twenty-eight lids were placed in one box, and empty holes in the racks were covered with aluminium foil to prevent light penetration. Then, two surface-sterilized Arabidopsis seeds were placed on the agar surface of each lid. The boxes were covered with plastic wrap and the seeds cold stratified at 4 °C for 3 days. Next, they were placed in a growth chamber under 8/16 h light/dark cycles at 22 °C, light intensity of 120 µE and 55% relative humidity. After 7 days, excess seedlings were removed to keep one seedling per hole, and the liquid medium was changed gradually to the standard growth solution as follows. On day 8, 30% of the germination medium was replaced with the standard growth solution, on day 9, 50% of the germination medium was replaced, and on day 10 the germination medium was entirely replaced by the standard growth medium. The plastic wrap was punctured on day 14 for the seedlings to adapt to the humidity in the chamber and completely removed after 17 days. The plants were allowed to grow for 6 weeks, with weekly solution changes before harvesting.

### Lipid extraction methods

Before the lipid extraction, all plant samples were ground in liquid nitrogen using mortar and pestle. Then, they were homogenized by cryomilling (Precellys 24, Bertin Technologies) for two consecutive 45 s intervals with a 30 s pause in between at 6100 rpm and a temperature of –10 °C with respective extraction solvents.

#### ***Welti ***et al***. method ***[18]***—M1***

The method by Welti et al. [18] is a multi-step extraction procedure. Plant material was homogenized by cryo-milling as described above with 1 ml of 2-propanol containing 0.01% butylated-hydroxy-toluene (BHT). The samples were heated up to 75 °C under constant shaking at 1400 rpm for 15 min. Next, they were cooled down to room temperature, and 0.5 ml CHCl_3_ and 0.2 ml water were added to each tube. The samples were incubated at room temperature under constant shaking at 1400 rpm for 1 h and centrifuged at 1,300 rpm for 15 min. The supernatant was carefully separated, and the samples were re-extracted with 0.3 ml of CHCl_3_/MeOH (2:1) with 0.01% BHT four times with 30 min incubation and 15 min centrifugation each time. The combined extracts were washed once with 0.4 ml 1 M potassium chloride (KCl) and once with 0.7 ml water. Finally, the solvents were evaporated by a vacuum concentrator until completely dry.

#### ***Hummel ***et al***. method ***[24]***—M2***

The method by Hummel et al. [24] is a biphasic extraction method where the upper organic phase contains the lipids, and the lower phase contains polar and semi-polar metabolites. The plant material was homogenized by cryomilling with 1 ml of a homogeneous mixture of MeOH: methyl-tert-butyl-ether (1:3). The samples were incubated for 10 min under shaking at 4 °C followed by a 10 min incubation in an ultrasonication bath at room temperature. Then, 500 µl of a homogeneous mixture of water and MeOH (3:1) was added to each tube, vortexed and centrifuged at 13,200 rpm for 15 min at room temperature. This step leads to phase separation. Next, the upper organic phase containing the lipids was transferred to a fresh tube, and the solvents evaporated in a vacuum concentrator until completely dry.

#### ***Burgos ***et al***. method ***[27]***—M3***

The protocol by Burgos et al. [27] details a rapid and simple method using an extraction temperature below room temperature. The plant material was homogenized by cryo-milling in 1 ml of a CHCl_3_/MeOH/water (1:2.5:1) mixture. The samples were incubated for 30 min at 4 °C before a 15 min centrifugation step at 13,200 rpm and 4 °C. The organic phase was transferred into a fresh tube and evaporated in a vacuum concentrator until completely dry.

#### ***Shiva ***et al***. method ***[23]***—M4***

Shiva et al. [23] modified the multi-step protocol published by Welti et al. [18] to a single-step extraction method with a 24 h extraction period. Plant material was homogenized by cryo-milling [23] with 400 µl of 2-propanol containing 0.01% BHT. The samples were heated at 75 °C for 15 min while shaking at 1,400 rpm. Then, they were allowed to reach room temperature, and 1.2 ml of a mixture of CHCl_3_/MeOH/water (30/41.5/3.5, v/v/v) was added to each sample. The samples were incubated at 25 °C for 24 h while shaking at 300 rpm. Next, the solvent was separated from the remaining sample and dried down using a vacuum concentrator.

### Liquid chromatography/ mass spectrometry (LC–MS) analysis of lipids

#### Chromatographic separation of lipids

The dried lipid extracts were re-suspended in 200 µl of butanol (BuOH) /MeOH (1:1) with 10 mM ammonium formate and subjected to LC–MS analysis as reported previously by Hu et al. [56] and described in brief below. The lipid extracts were transferred to vials and placed in the autosampler tray which was set at 12 °C; then they were separated by loading 5 µl aliquots onto an InfinityLab Poroshell 120 EC-C18 2.1 × 100 mm (2.7-Micron particle size) column (Agilent, USA) operated at 55 °C using an Agilent 1290 HPLC system and a flow rate of 0.26 ml/min. Elution was performed over a 30 min binary gradient consisting of acetonitrile (ACN)-water (60:40, v/v) and isopropanol (IPP)-ACN (90/10, v/v) both containing 10 mM ammonium formate as eluent A and B respectively. The gradient used was set to first a 0–1.5 min isocratic elution with 32% B which was then increased to 45% B from 1.5 to 4 min, then to 52% B from 4 to 5 min followed by an increase to 58% B from 5 to 8 min. Next, the gradient was increased to 66% B from 8 to 11 min followed by an increase to 70% B from 11 to 14 min and an increase to 75% B from 14 to 18 min. Then, from 18 to 21 min B was increased to 97% and B was maintained at 97% from 21 to 25 min. Finally, solvent B was decreased to 32% from 25 to 25.10 min, and B was maintained at 32% for another 4.9 min for column re-equilibration [56].

#### Analysis of lipids by mass spectrometry

Lipids were analyzed by ESI–MS/MS using a 6545-series quadrupole-time of flight mass spectrometer (Agilent) using both full scan mode and auto MS/MS mode. Data were accumulated in both positive and negative modes using a mass range of 200–1700 m*/z* in full scan mode and 100–1700 m/z in Auto MS/MS mode. The MS1 acquisition rate was 1 spectrum/s with 1000 ms/spectrum while the MS/MS acquisition rate was 3 spectra/s with 333.3 ms/spectrum. The isolation width in Auto MS/MS mode was medium, precursors/cycle was 20, collision energy was fixed at 10, 20 and 40 and the absolute threshold for MS/MS was set at 5 counts and the relative threshold at 0.01%.

### Data processing

The raw LC–MS data were converted into analysis base file (ABF) format using the Reifycs file converter and processed through the open-source software MS-DIAL [57]. The parameters were as follows: MS1 tolerance = 0.01 Da, MS2 tolerance = 0.025 Da, Retention time = 0–30 min, MS1 mass range = 0–1700 Da and minimum peak height = 10,000 amplitude. The peaks were aligned to a quality control sample with a retention time tolerance of 1 min and MS1 tolerance of 0.025 min. All other parameters were kept at the default values for conventional LC/MS or data-dependent MS/MS data processing. MS-DIAL output consisting of the peak area of detected compounds was analyzed using Microsoft Excel. Annotation of detected lipids was performed by searching their mass/charge ratios against the accurate masses of a compiled list of lipids (< 0.01 Da mass error), matching the tandem mass spectrometric data of the auto MS/MS mode with the fragment library in MS-DIAL internal lipid database [57] and the respective retention time patterns. The quality control sample was prepared by combining 10 µl of each sample extract prepared for LC–MS analysis.

### Statistical analysis

Five technical replicates were prepared from each Arabidopsis sample and analyzed. The peak area of the full scan mode LC–MS features extracted by MS-DIAL were normalized to the fresh weight of each sample, log-transformed, auto-scaled and statistically analyzed by the freely available online software, MetaboAnalyst (www.metaboanalyst.ca/MetaboAnalyst) [58]. To determine statistically significant differences, one-way analysis of variance (ANOVA) followed by Tukey’s honestly significant difference (HSD) post hoc test (p < 0.05) was carried out using MetaboAnalyst [58].

### Repeatability

To test the repeatability, i.e. the ability of each method to produce similar results from five replicates under the same conditions, we compared the distribution of the coefficient of variation (CV) of peak area measurements among the five technical replicates for each tissue prepared with the different extraction protocols. The CVs of peak area measurements within five technical replicates were calculated by dividing the standard deviation of the peak area measurements of the five replicates by the mean. The CV values were presented as percentage [48]. Then, the percentage of lipids with a CV below 20% out of the total number of lipids detected from each tissue was also calculated. Subsequently, the values were compared to evaluate the repeatability of each method [43]. The repeatability was further evaluated by hierarchical cluster analysis using MetaboAnalyst (www.metaboanalyst.ca/MetaboAnalyst) [58], which shows the clustering results in the form of a dendrogram. Two parameters were used by MetaboAnalyst for hierarchical cluster analysis. One parameter is the similarity measure using Euclidean distance, Pearson’s correlation and Spearman’s rank correlation and the other parameter is clustering algorithms using average linkage, complete linkage, single linkage and Ward’s linkage [58]. In addition to the dendrograms heat maps were generated to aid visualization of the results [58] with each coloured cell in a heat map corresponding to a value in the data table.

## Supplementary Information

**Additional file 1.** Lipids identified from each of the Arabidopsis tissues analyzed and the experimental data used in the study.

**Additional file 2.** Bar graphs and heat maps showing the differences in extractability of individual lipid classes from flowers, roots, siliques, stems, seedlings and seeds of Arabidopsis.

**Additional file 3.** Germination medium and standard growth solution used in the study for hydroponic growth of Arabidopsis to obtain root material.

## Data Availability

All datasets generated for this study are included in the article/additional material.

## Abbreviations

PC: Phosphatidylcholine
PE: Phosphatidylethanolamine
PG: Phosphatidylglycerol
PI: Phosphatidylinositol
PS: Phosphatidylserine
PA: Phosphatidic acid
LPC: Lysophosphatidylcholine
LPE: Lysophosphatidylethanolamine
Cer-AP: Ceramides
HexCer: Hexsolyceramides
CL: Cardiolipins
DGDG: Digalactosyldiacylglycerol
MGDG: Monogalactosylmonoacylglycerol
SQDG: Sulfoquinovosyldiacylglycerol
DAG: Diacylglycerol
TAG: Triacylglycerol
CHCl_3_: Chloroform
MeOH: Methanol
BuOH: Butanol
BHT: Butylated-hydroxy-toluene
KCl: Potassium chloride
HCl: Hydrochloric acid
Q-TOF: Quadrupole time-of-flight
