# Supplementary material for: A comprehensive comparison of four methods for extracting lipids from Arabidopsis tissues
Source: Plant Methods. 2020 Dec 3;16:155. doi: 10.1186/s13007-020-00697-z (PMC7713330; doi:10.1186/s13007-020-00697-z)
Supplement: Supplementary file 2 — Additional file 2. Bar graphs and heat maps showing the differences in extractability of individual lipid classes from flowers, roots, siliques, stems, seedlings and seeds of Arabidopsis. [file 13007_2020_697_MOESM2_ESM.pdf]

Fig S1a. Comparison of the extractability of individual lipid classes from flowers of *Arabidopsis thaliana* by the four lipid extraction methods. Bars show the average peak area of the lipids belonging to a class normalized to the fresh weight of flower tissue samples (mean±SD, n=5). Different letters above bars of the same tissue indicate significant differences ($p < 0.05$) in total lipid extractability of the four methods by ANOVA test followed by Tukey's test. **b.** Heat map of the lipid classes extracted from flowers by the four protocols (n=5). Red colour bars (M1): Welti et al., Green colour bars (M2): Hummel et al., Dark blue colour bars (M3): Burgos et al., Light blue colour bars (M4): Shiva et al.

a

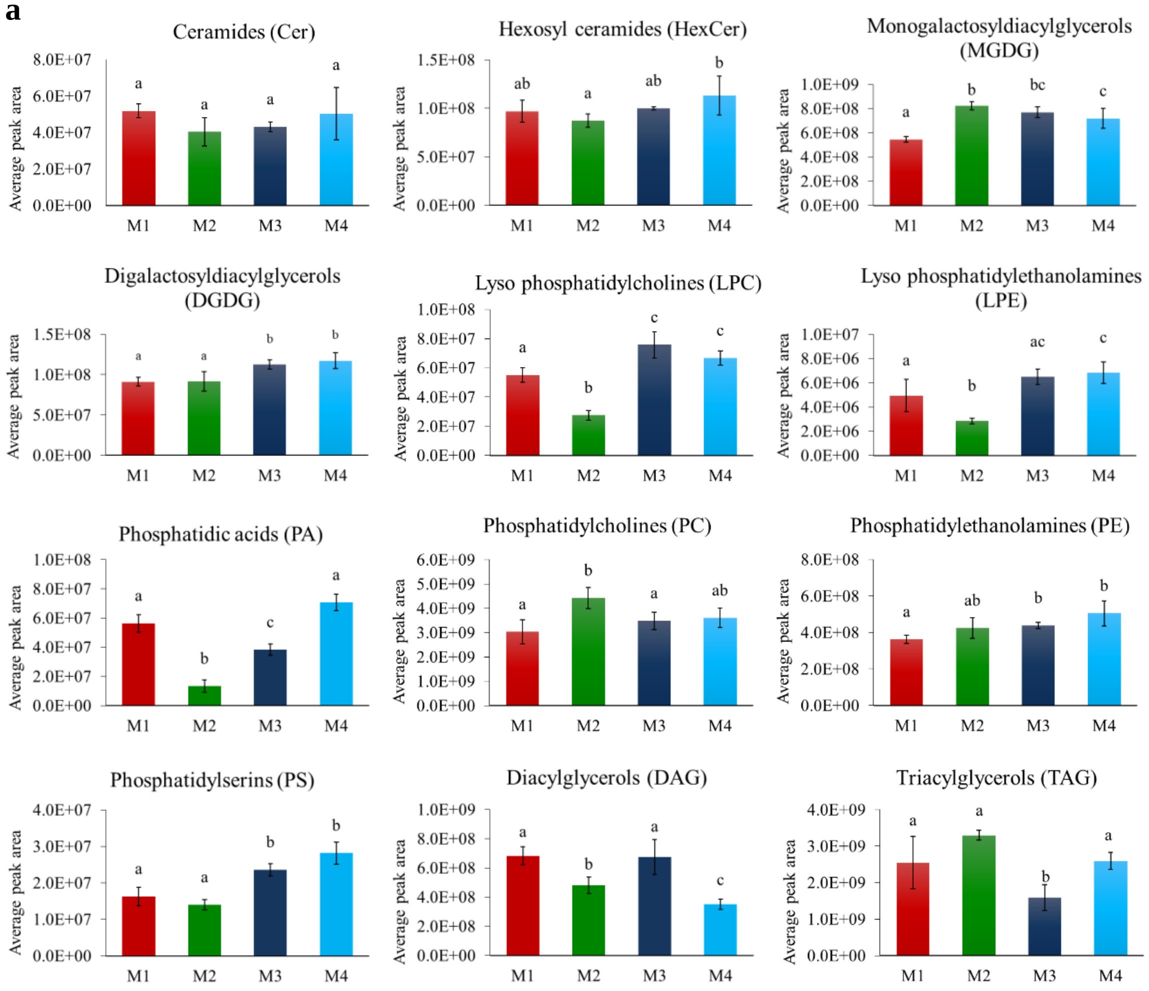

M1: Welti et al. M2: Hummel et al. M3: Burgos et al. M4: Shiva et al.

b

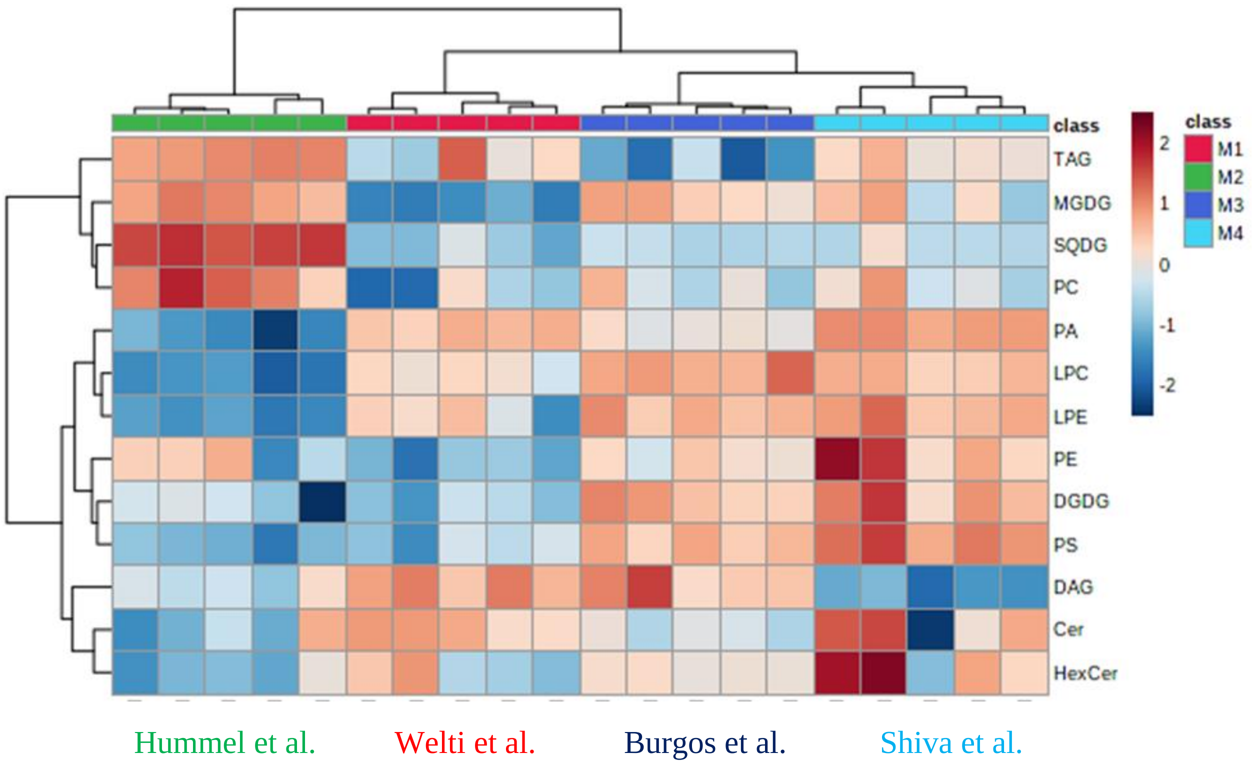
class
TAG
MGDG
SQDG
PC
PA
LPC
LPE
PE
DGDG
PS
DAG
Cer
HexCer
2
1
0
-1
-2
class
M1
M2
M3
M4
Hummel et al.
Welti et al.
Burgos et al.
Shiva et al.

Fig S2a. Comparison of the extractability of individual lipid classes from siliques of *Arabidopsis thaliana* by the four lipid extraction methods. Bars show the average peak area of the lipids belonging to a class normalized to the fresh weight of siliques (mean±SD, n=5). Different letters above bars of the same tissue indicate significant differences ($p < 0.05$) in total lipid extractability of the four methods by ANOVA test followed by Tukey's test. **b.** Heat map of the lipid classes extracted from siliques by the four protocols (n=5). Red colour bars (M1): Welti et al., Green colour bars (M2): Hummel et al., Dark blue colour bars (M3): Burgos et al., Light blue colour bars (M4): Shiva et al.

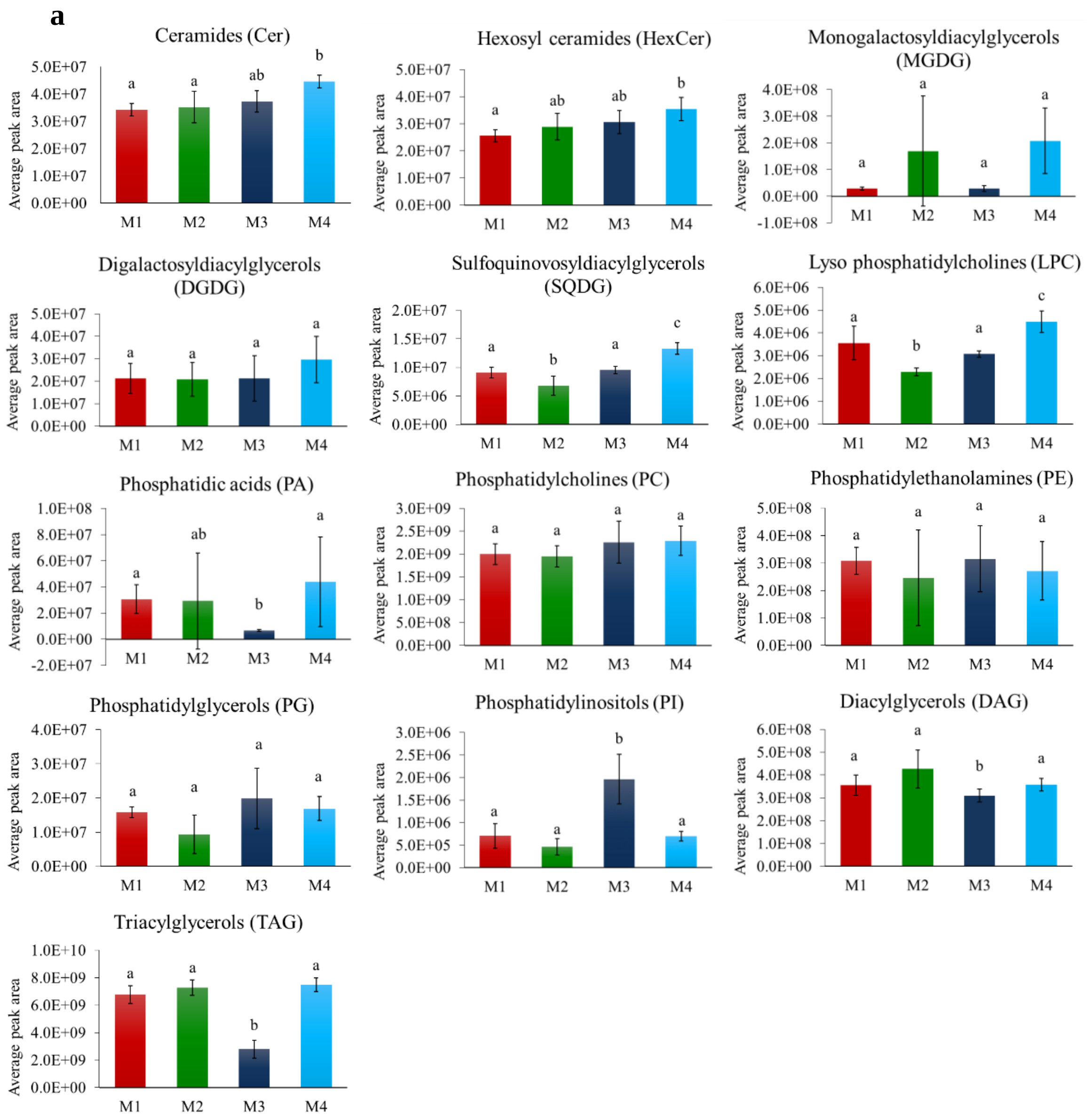

M1: Welti et al. M2: Hummel et al. M3: Burgos et al. M4: Shiva et al.

b

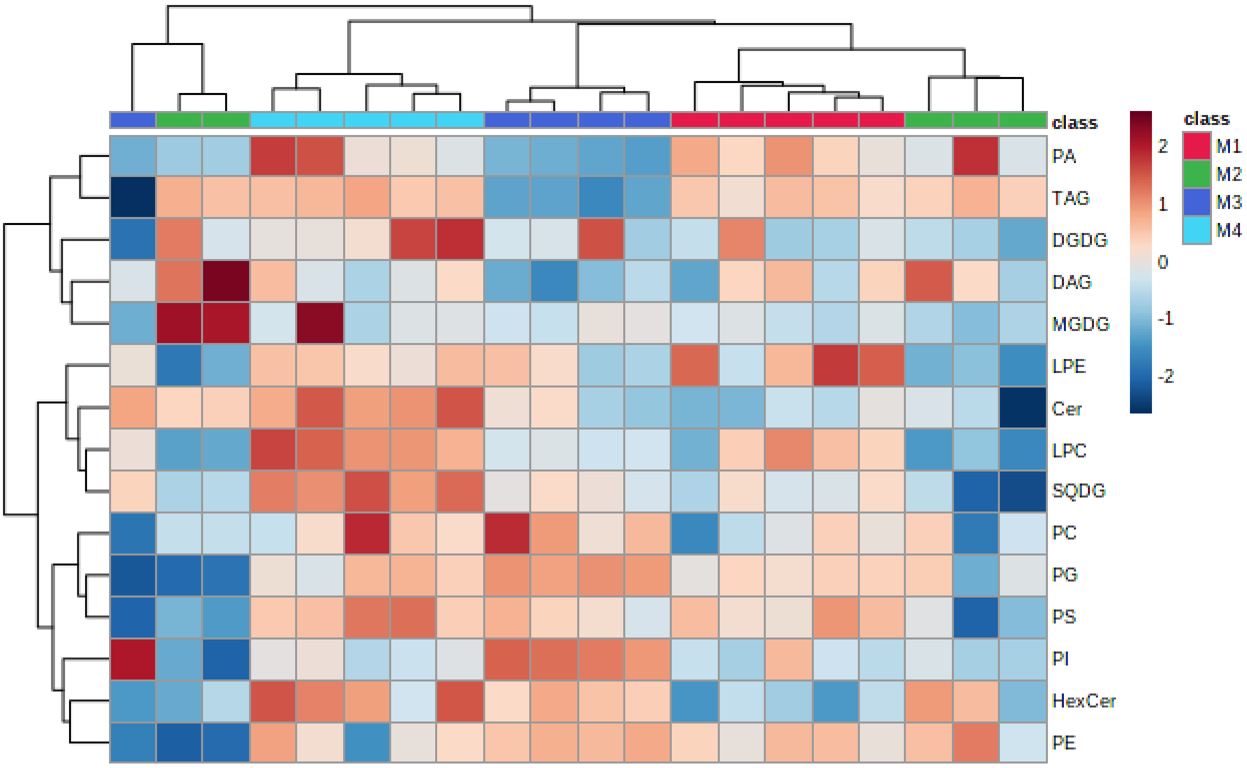

Hummel et al. Welti et al. Burgos et al. Shiva et al.

Fig S3a. Comparison of the extractability of individual lipid classes from seeds of *Arabidopsis thaliana* by the four lipid extraction methods. Bars show the average peak area of the lipids belonging to a class normalized to the fresh weight of seed sample (mean±SD, n=5). Different letters above bars of the same tissue indicate significant differences ($p < 0.05$) in total lipid extractability of the four methods by ANOVA test followed by Tukey's test. **b.** Heat map of the lipid classes extracted from seed samples by the four protocols (n=5). Red colour bars (M1): Welti et al., Green colour bars (M2): Hummel et al., Dark blue colour bars (M3): Burgos et al., Light blue colour bars (M4): Shiva et al.

a

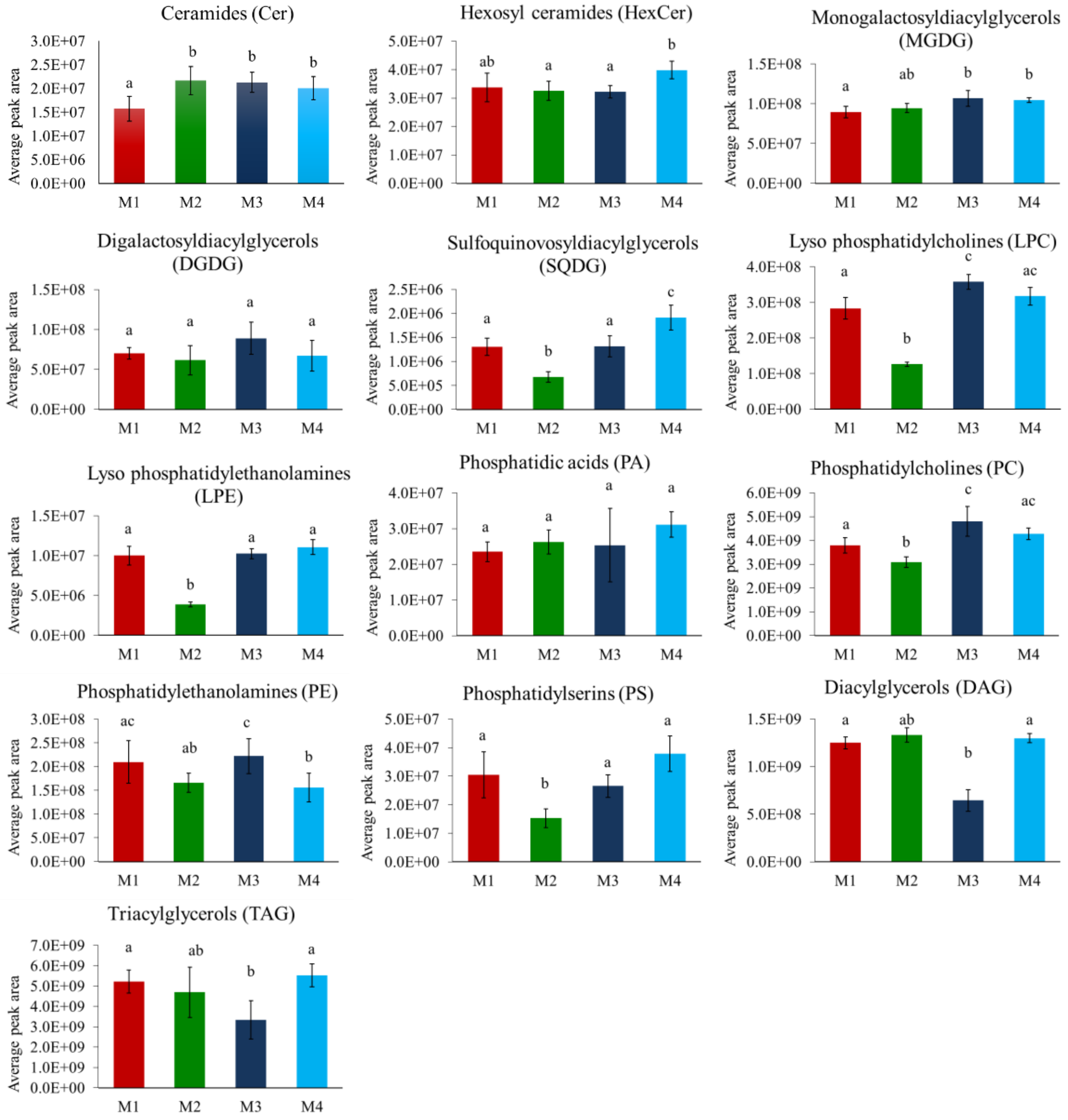

M1: Welti et al.   M2: Hummel et al.   M3: Burgos et al.   M4: Shiva et al.

b

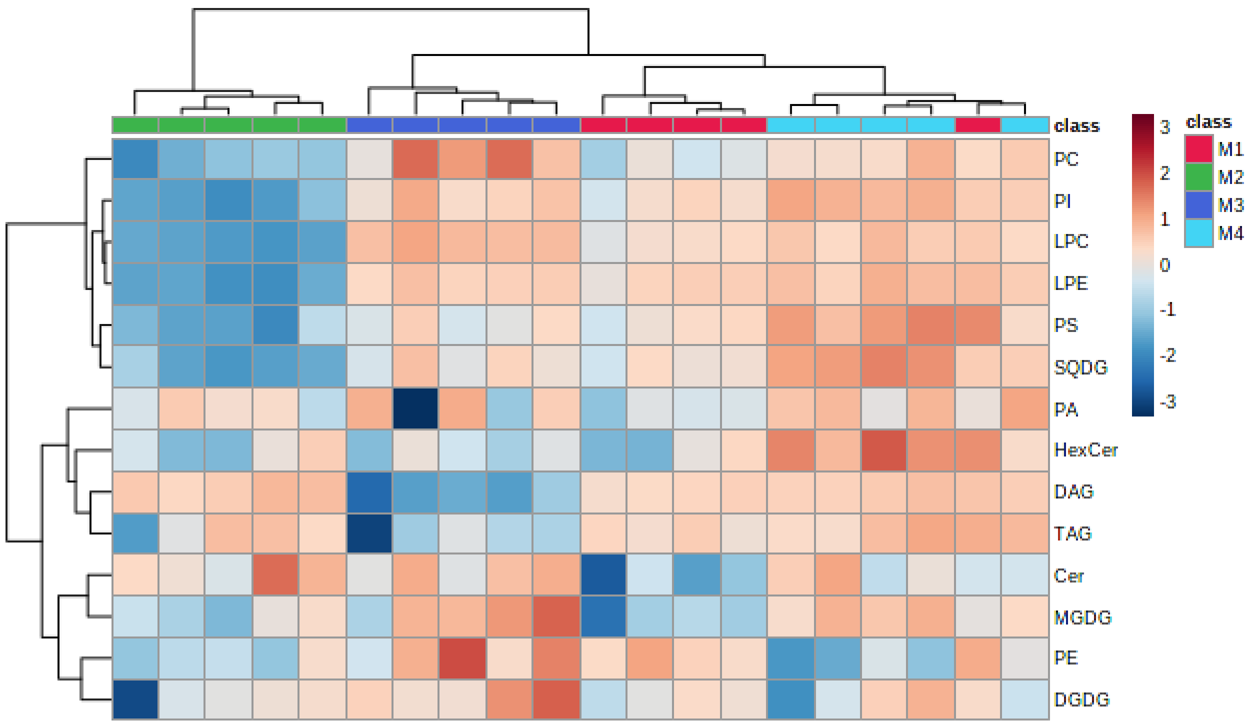

M1: Welti et al. M2: Hummel et al. M3: Burgos et al. M4: Shiva et al.

Fig S4a. Comparison of the extractability of individual lipid classes from seedlings of *Arabidopsis thaliana* by the four lipid extraction methods. Bars show the average peak area of the lipids belonging to a class normalized to the fresh weight of the seedling sample (mean±SD, n=5). Different letters above bars of the same tissue indicate significant differences ($p < 0.05$) in total lipid extractability of the four methods by ANOVA test followed by Tukey's test. **b.** Heat map of the lipid classes extracted from seedling samples by the four protocols (n=5). Red colour bars (M1): Welti et al., Green colour bars (M2): Hummel et al., Dark blue colour bars (M3): Burgos et al., Light blue colour bars (M4): Shiva et al.

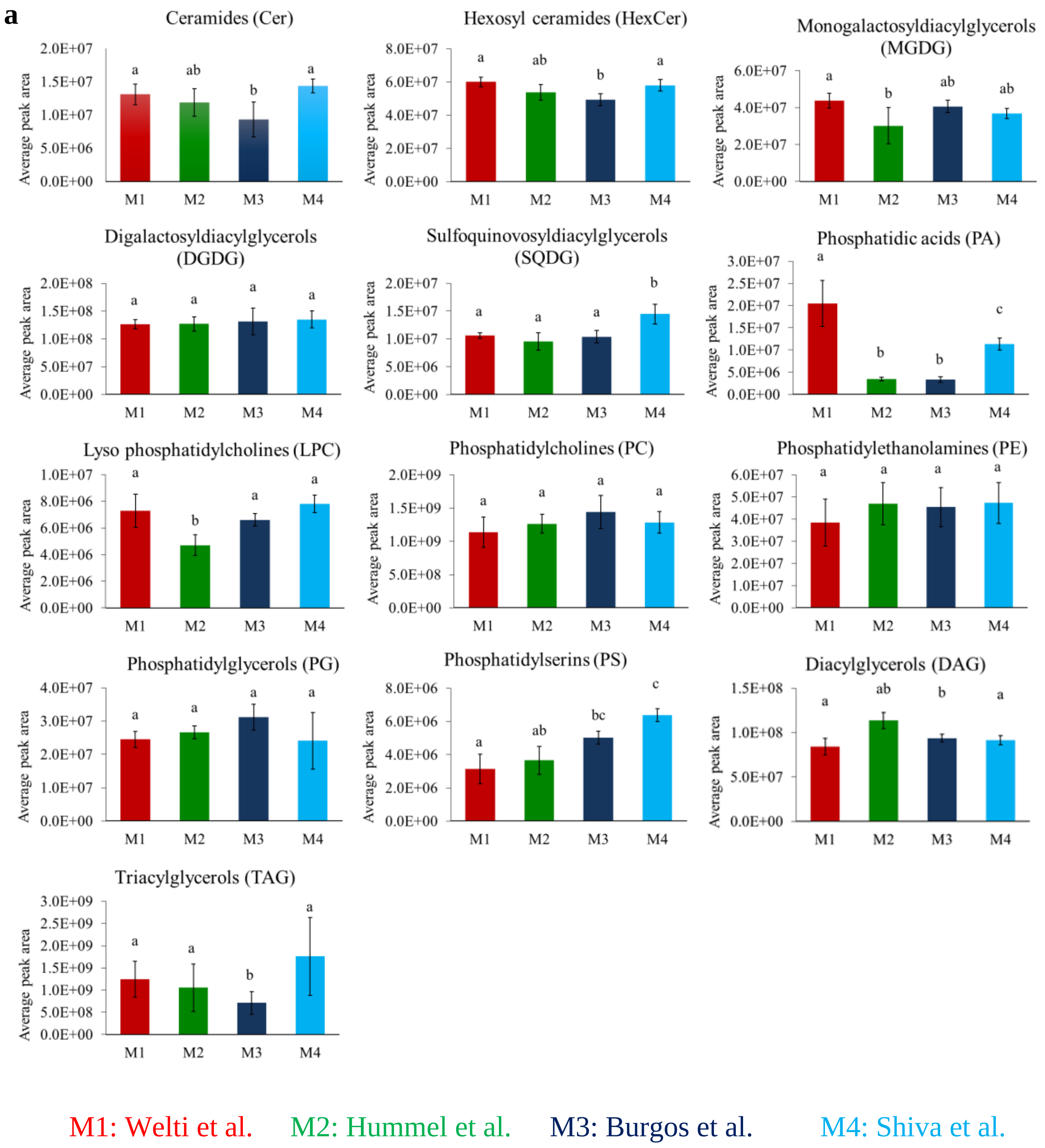

b

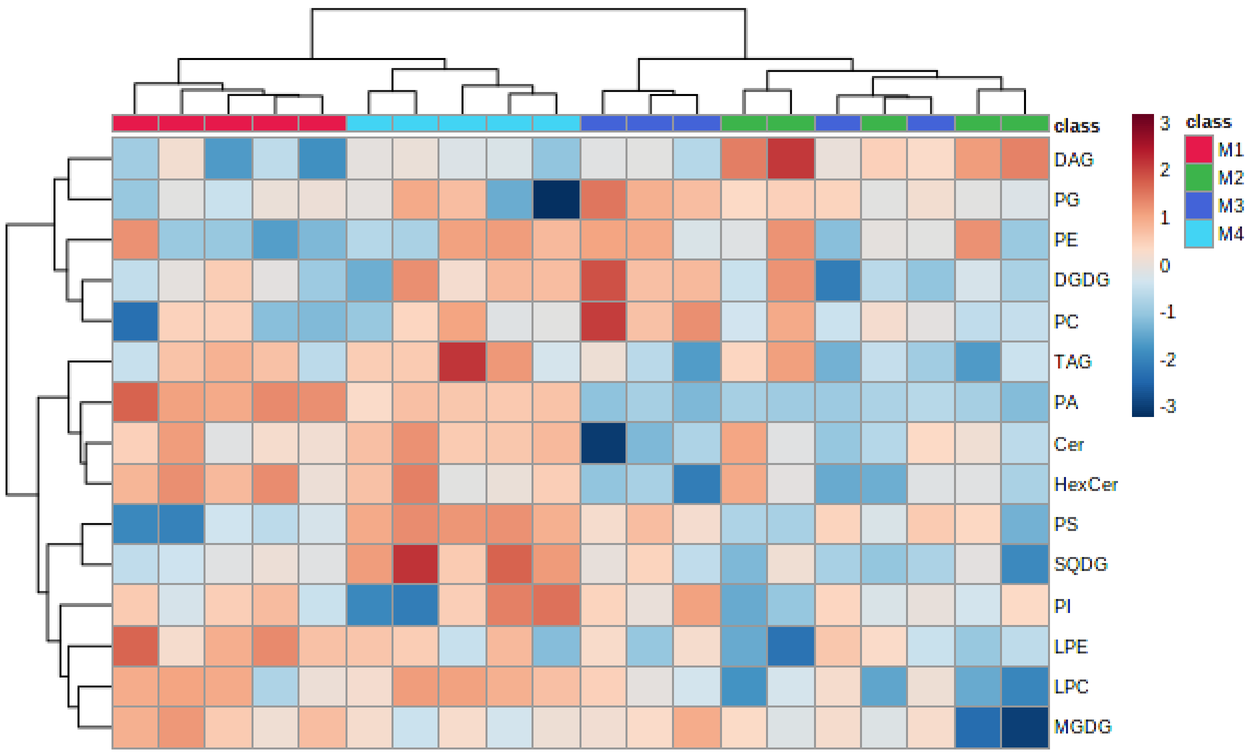

M1: Welti et al. M2: Hummel et al. M3: Burgos et al. M4: Shiva et al.

Fig S5a. Comparison of the extractability of individual lipid classes from stems of *Arabidopsis thaliana* by the four lipid extraction methods. Bars show the average peak area of the lipids belonging to a class normalized to the fresh weight of stem sample (mean±SD, n=5). Different letters above bars of the same tissue indicate significant differences ($p < 0.05$) in total lipid extractability of the four methods by ANOVA test followed by Tukey's test. **b.** Heat map of the lipid classes extracted from stem samples by the four protocols (n=5). Red colour bars (M1): Welti et al., Green colour bars (M2): Hummel et al., Dark blue colour bars (M3): Burgos et al., Light blue colour bars (M4): Shiva et al.

a

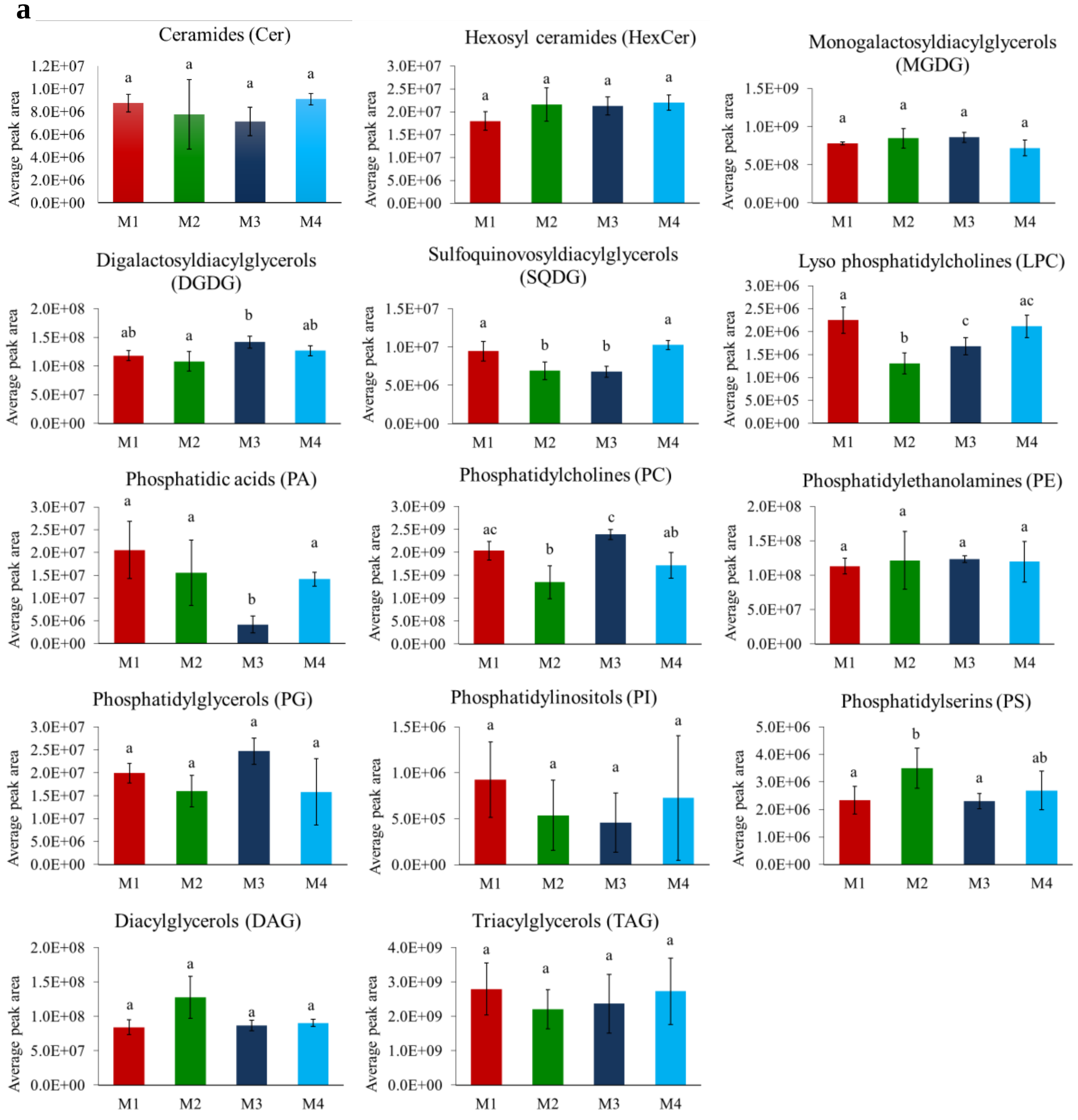

M1: Welti et al. M2: Hummel et al. M3: Burgos et al. M4: Shiva et al.

b

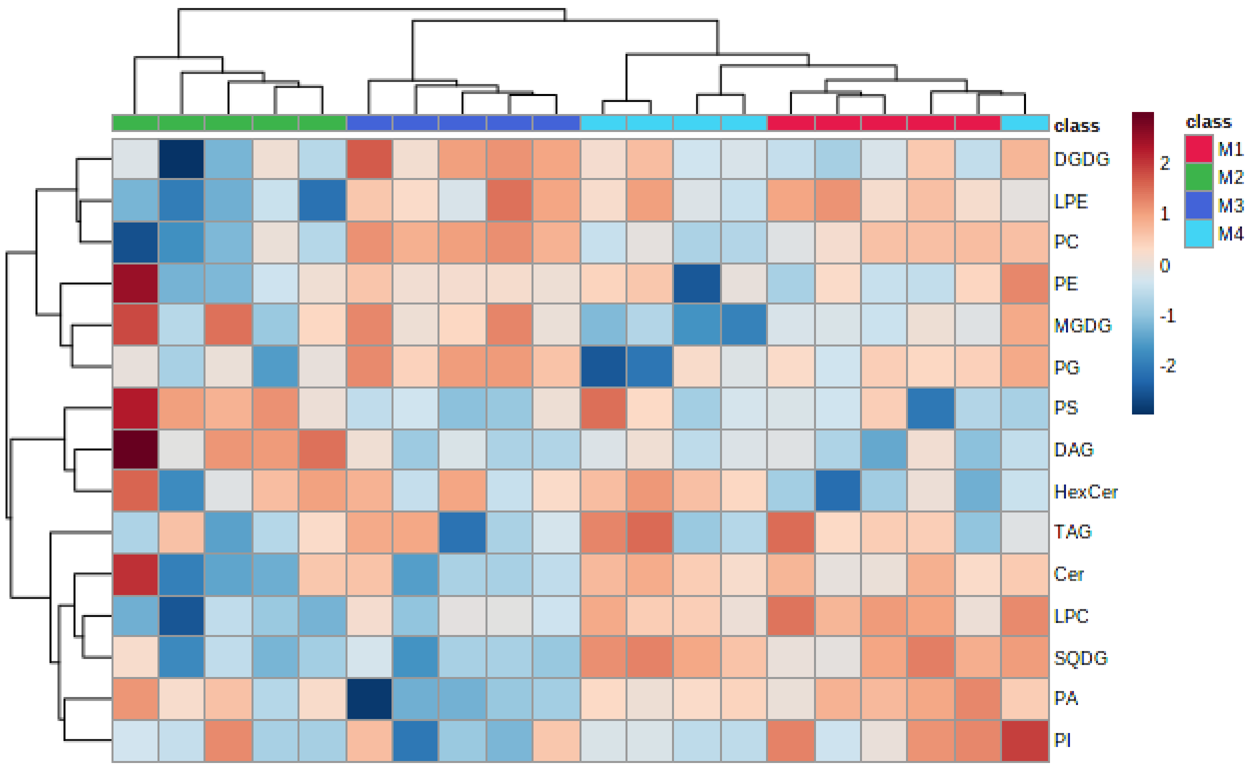

M1: Welti et al.   M2: Hummel et al.   M3: Burgos et al.   M4: Shiva et al.

Fig S6a. Comparison of the extractability of individual lipid classes from roots of *Arabidopsis thaliana* by the four lipid extraction methods. Bars show the average peak area of the lipids belonging to a class normalized to the fresh weight of root sample (mean±SD, n=5). Different letters above bars of the same tissue indicate significant differences ($p < 0.05$) in total lipid extractability of the four methods by ANOVA test followed by Tukey's test. **b.** Heat map of the lipid classes extracted from root samples by the four protocols (n=5). Red colour bars (M1): Welti et al., Green colour bars (M2): Hummel et al., Dark blue colour bars (M3): Burgos et al., Light blue colour bars (M4): Shiva et al.

a

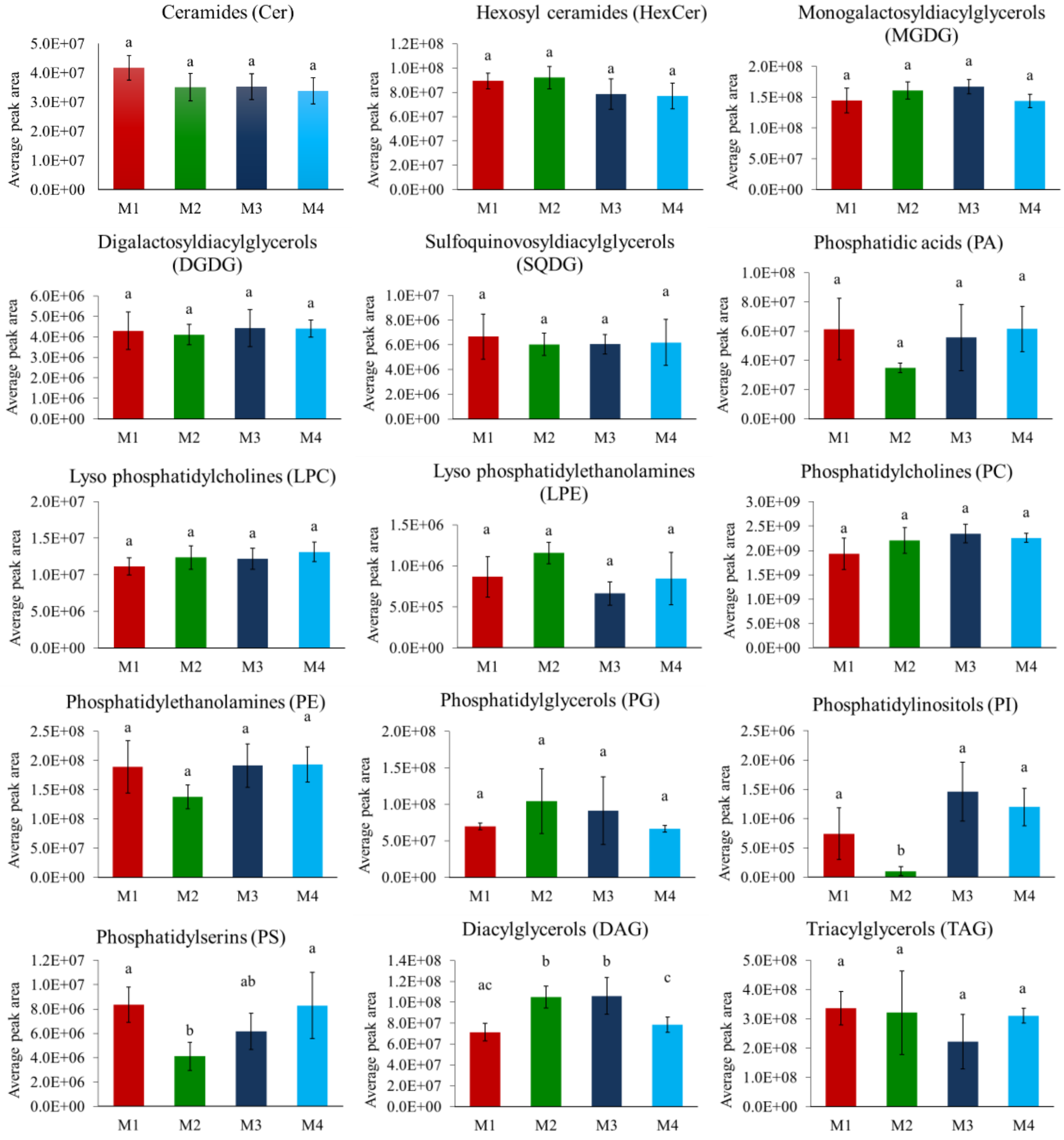

M1: Welti et al.   M2: Hummel et al.   M3: Burgos et al.   M4: Shiva et al.

b

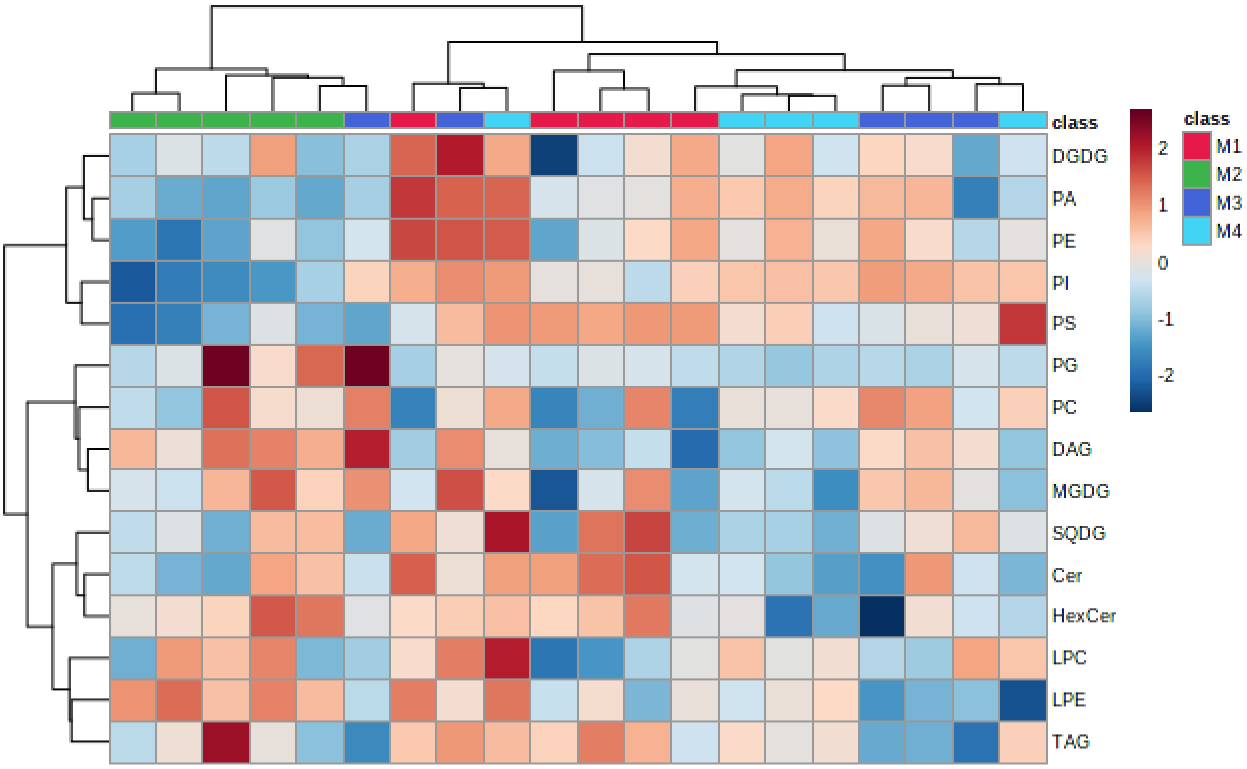

M1: Welti et al.   M2: Hummel et al.   M3: Burgos et al.   M4: Shiva et al.
