# Supplementary material for: A comprehensive comparison of four methods for extracting lipids from Arabidopsis tissues
Source: Plant Methods. 2020 Dec 3;16:155. doi: 10.1186/s13007-020-00697-z (PMC7713330; doi:10.1186/s13007-020-00697-z)
Supplement: Supplementary file 3 — Additional file 3. Germination medium and standard growth solution used in the study for hydroponic growth of Arabidopsis to obtain root material. [file 13007_2020_697_MOESM3_ESM.pdf]

The recipes for the germination medium and standard growth medium by Conn, S.J. et al. Protocol: optimising hydroponic growth systems for nutritional and physiological analysis of *Arabidopsis thaliana* and other plants. *Plant Methods* **9**, 4 (2013). https://doi.org/10.1186/1746-4811-9-4.

Germination medium

**Macronutrients**	**Formula weight**	**g to make 1 L stock**	**Stock Concentration (M)**	**Volume of stock (mL) for 1L**	**Final concentration (mM)**
NH_4NO_3	80	80	1	**0**	0
KNO_3	101.1	101.1	1	**0**	0
$CaCl_2$	1M Solution		1	**0.75**	0.75
KCl	74.55	74.55	1	**1**	1
$Ca(NO_3)_2$•$4H_20$	236.1	94.4	0.4	**0.625**	0.25
$MgSO_4$•$7H_20$	246.5	98.6	0.4	**2.5**	1
KH_2PO_4	136.1	13.61	0.1	**2**	0.2
**Micronutrients**	**Formula weight**	**g to make 1 L stock**	**Stock Concentration (mM)**	**Volume of stock (mL) for 1L**	**Final concentration (uM)**
NaFe(III)EDTA	367.1	18.4	50	**1**	50
H_3BO_3	61.8	3.09	50	**1**	50
$MnCl_2$•$4H_20$	197.9	0.99	5	**1**	5
$ZnSO_4$•$7H_20$	287.5	2.875	10	**1**	10
$CuSO_4$•$5H_20$	249.7	0.125	0.5	**1**	0.5
Na_2MoO_3	242	0.0245	0.1	**1**	0.1
**pH with NaOH to 5.6**					
**0.7% Agar**	**7g per 1000ml**				
Macronutrients			Micronutrients		
**Final concentration (mM)**		**Activity**	**Final concentration (mM)**		**Activity**
K	1.2	4.79	Fe	0.01	25 pM
Ca	1	1.05	Mn	0.005	23nM
Mg	1	1.03	Zn	0.01	50 uM
NH_4	0	1.72	Cu	0.0005	23 nM
Cl	2.51	3.19	Mo	0.0001	31 nM
NO_3	0.5	7.75			
SO_4	1.0105	0.893			
PO_4	0.2	1.8 pM			
Na	0.1012	1.38			

Standard growth solution

**Macronutrients**	**FW**	**g to make 1 L stock**	**Stock concentration (M)**	**Vol of stock (mL) for 1L**	**Final concentration (mM)**
NH_4NO_3	80	80	1	**2**	2
KNO_3	101.1	101.1	1	**3**	3
$CaCl_2$	1M solution		1	**0.1**	0.1
KCl	74.55	74.55	1	**2**	2
$Ca(NO_3)_2$•$4H_20$	236.1	94.4	0.4	**5**	2
$MgSO_4$•$7H_20$	246.5	98.6	0.4	**5**	2
KH_2PO_4	136.1	13.61	0.1	**6**	0.6
NaCl	58.44	58.44	1	**1.5**	1.5
**Micronutrients**	**FW**	**g to make 1 L stock**	**Stock concentration (mM)**	**Vol of stock (mL) for 1L**	**Final concentration (uM)**
NaFe(III)EDTA	367.1	18.4	50	**1**	50
H_3BO_3	61.8	3.09	50	**1**	50
$MnCl_2$•$4H_20$	197.9	0.99	5	**1**	5
$ZnSO_4$•$7H_20$	287.5	2.875	10	**1**	10
$CuSO_4$•$5H_20$	249.7	0.125	0.5	**1**	0.5
Na_2MoO_3	242	0.0245	0.1	**1**	0.1
**pH with NaOH to 5.6**					
Macronutrients			Micronutrients		
**Final concentration (mM)**		**Activity**	**Final concentration (mM)**		**Activity**
K	5.6	4.79	Fe	0.01	25 pM
Ca	2.1	1.05	Mn	0.005	23nM
Mg	2	1.03	Zn	0.01	50 uM
NH_4	2	1.72	Cu	0.0005	23 nM
Cl	3.71	3.19	Mo	0.0001	31 nM
NO_3	9	7.75			
SO_4	2.0105	0.893			
PO_4	0.6	1.8 pM			
Na	1.5502	1.38			
